# Profiling extracellular vesicles from cerebrospinal fluid for classification of intradural spinal tumors

**DOI:** 10.1038/s41598-025-23190-w

**Published:** 2025-10-20

**Authors:** Amanda Salviano-Silva, Ines Stevic, Christian Mende, Rudolph Reimer, Cecile L. Maire, Marius M. Mader, Lasse Dührsen, Katrin Lamszus, Manfred Westphal, Sven O. Eicker, Franz L. Ricklefs

**Affiliations:** 1 Department of Neurosurgery, University Medical Center Hamburg-Eppendorf, Martinistrasse 52, 20246 Hamburg, Germany; 2 Department of Neurosurgery, Friedrich-Ebert-Krankenhaus, Neumuenster, Germany; 3 Leibniz Institute for Experimental Virology, Hamburg, Germany; 4 Department of Spine and Scoliosis Surgery, Lubinus Clinicum, Kiel, Germany

**Keywords:** Extracellular vesicles, Biomarkers, Intradural spinal tumors, CSF, Liquid biopsies, Cancer screening, Tumour biomarkers, Nanotechnology in cancer

## Abstract

Extracellular vesicles (EVs) transport biomolecules that could serve as biomarkers for disease diagnosis and monitoring. The clinical utility of EVs derived from cerebrospinal fluid (CSF) in patients with intradural spinal tumors (IST) has not yet been investigated. Here, we obtained EVs from CSF of adult patients with intraspinal ependymoma (n = 9), meningioma (n = 9), hemangioma (n = 4) and schwannian tumors (n = 7), as well as comparison group (‘CG’, normal pressure hydrocephalus, n = 7), by ultrafiltration. CSF-EVs were characterized by electron microscopy and nanoparticle tracking analysis. EV populations according to the presence of tetraspanins (CD9, CD63, CD81) were measured by imaging flow cytometry (IFCM). CD81^+^ EVs were more prevalent in the comparison group, meningioma, ependymoma WHO grade 2, and hemangioma, whereas CD9^+^ EVs were predominant in ependymoma grade 1 and Schwannian tumors. CD63^+^ EVs per milliliter/CSF differed between ependymoma WHO grades 1 and 2 (FC = 24.6, AUC = 90%, *p* < 0.05). Based on results from a bead-based multiplex profiling, we selected ITGB1, CD44, CD133 and HLA-DR/DQ/DP for further phenotyping in CSF-EVs using IFCM, in combination with each tetraspanin as double-positive subpopulations. Compared to CG, CD44^+^ EVs were the most relevant population in CSF from IST patients, followed by ITGB1. Notable differences in absolute (EVs/mL CSF) and relative (percentages of CSF-EVs) levels were: CD44^+^/CD81^+^ for ependymoma grade 1 (FC = 196.5 and 34.5; *p* < 0.01) and grade 2 (%FC = 6.1, *p* < 0.05); CD44^+^/CD63^+^ for meningioma (abs. and %FC > 1000, *p* < 0.05); ITGB1^+^/CD81^+^ for hemangioma (%FC = 4.8, *p* < 0.05); and ITGB1^+^/CD9^+^ for schwannian tumors (abs.FC = 19.8, *p* < 0.01). In conclusion, we identified distinct EV subpopulations in the CSF of IST patients, potentially facilitating tumor classification.

**Supplementary Information:**

The online version contains supplementary material available at 10.1038/s41598-025-23190-w.

## Introduction

Extracellular vesicles (EVs), a diverse group of cell-derived membranous nanostructures, have emerged as a significant area of interest for disease diagnostics and therapeutics^1–3^. EVs are abundantly secreted by cancer cells into the tumor microenvironment (TME) and can be found in various bodily fluids such as blood, urine, and cerebrospinal fluid (CSF). The molecular components encapsulated in EVs (e.g., proteins, RNA, DNA, lipids) mirror the physiological and pathological states of their cells of origin^1,2^. This makes them effective carriers of disease-specific markers, particularly for tumors within the central nervous system (CNS) where direct access is limited^4^. In this context, the majority of studies with EVs in CNS tumors have been performed in brain cancers, where EV subpopulations have been identified as biomarkers for glioblastoma and meningioma^5–10^. Meanwhile, EVs in patients with tumors affecting the spinal cord (e.g., spinal meningiomas, schwannomas, and ependymomas) still lack investigation. Owing to their sensitive location, intradural spinal tumors (IST) pose significant challenges in terms of diagnosis, treatment, and monitoring. Currently, diagnosis largely relies on imaging techniques and invasive biopsy procedures, which carry risks of complications and may not fully capture the heterogeneity of the tumors^11^. The minimally invasive nature of obtaining EVs from biological fluids aligns well with the clinical need for safer, repeatable, and more sensitive diagnostic methods^1,4,12^.

In this study, we investigated the use of EVs as a biomarker source in the CSF of patients with IST. We performed membrane profiling of CSF-EVs using single-EV technologies and discuss the technological advancements and challenges associated with isolating and characterizing these vesicles from biological fluids. Our results highlight the transformative potential of EV biomarkers in improving the diagnosis, prognosis, and monitoring of ISTs.

## Results

### Characterization and differential levels of tetraspanins’ populations in CSF-EVs from IST patients

**Fig. 1:**
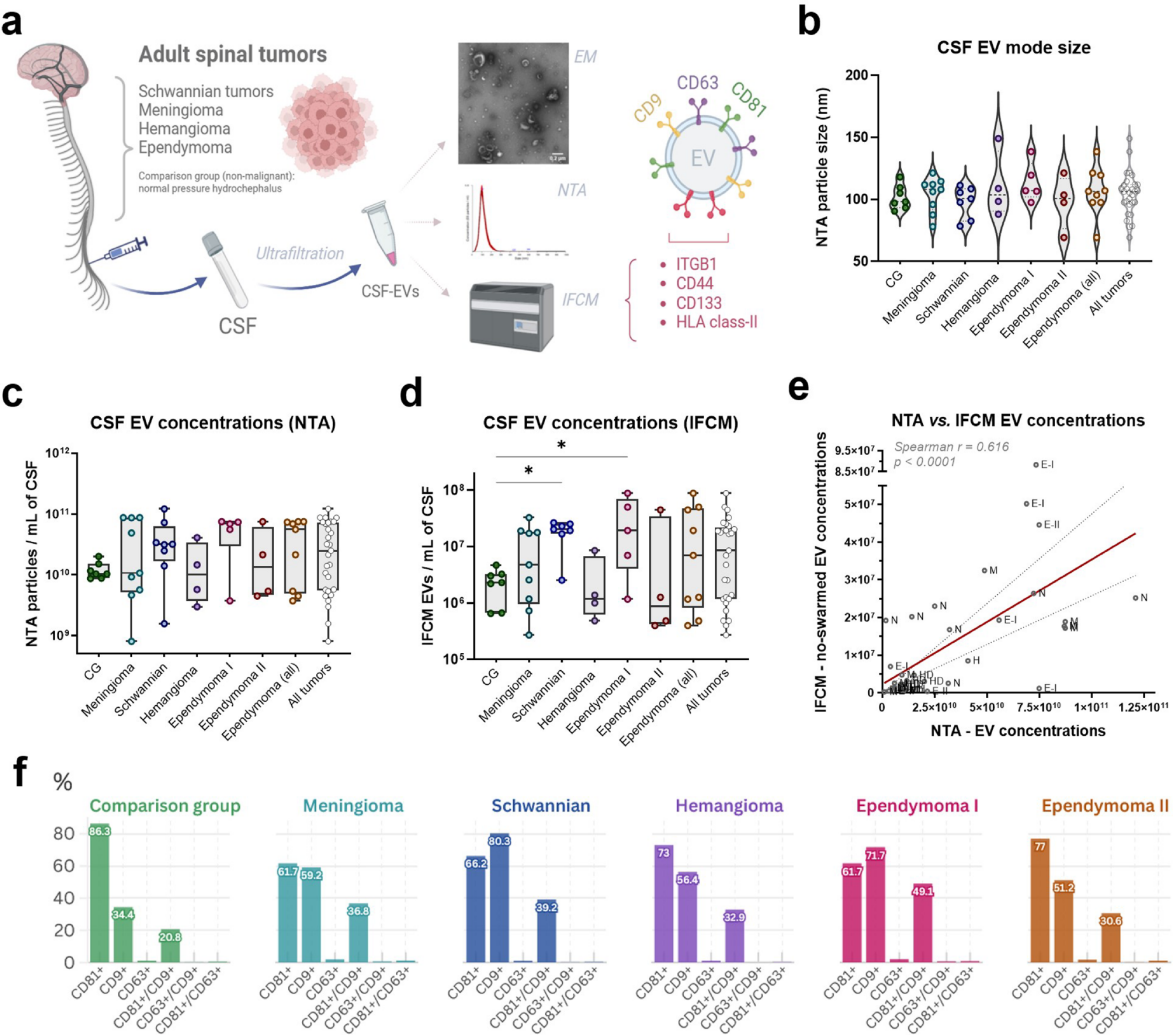
CSF-EV characterization and concentration levels. (**a**) Methodological workflow. CSF-derived EVs were isolated by filtration from adult patients suffering with spinal tumors and analyzed by (I) transmission electron microscopy (EM), where EVs presented a characteristic cup shape; (II) NTA, for evaluation of EV size and concentration (see Supp. Figure S1 and Supp. Table S1); and (III) IFCM, for quantification of EVs populations that are positive for tetraspanins (CD81, CD63 and CD9) and tumor-associated markers (ITGB1, CD44, CD133 and HLA class-II). Figure created with *Biorender.com*. (**b**) CSF-EVs showed a size mode range between 69.3 nm and 149 nm (see Supp. Table S1) and were not significantly different among patients’ groups (ANOVA test). (**c**) According to NTA, nanoparticle concentrations per milliliter of CSF were not significantly different in IST patients, when compared to comparison group (CG). (**d**) According to IFCM, most IST entities show similar non-swarmed EV levels, with exception of patients suffering with schwannian tumors and ependymoma WHO grade 1 patients, who respectively had 8.77- and 8.38-fold higher levels than CG (Kruskal–Wallis test; * = *p* <0 0.05). (**e**) Linear regression shows positive correlation between EV counts obtained with NTA and IFCM methods, despite the lower levels quantified by IFCM (Spearman r = 0.616, *p* < 0.0001). (**f**) Characterization of tetraspanin percentages (as single- and double-positive populations) in CSF EVs of each IST entity. Graphs created with *flourish.studio*.

We isolated EVs from the CSF of individuals without neuro-oncological disease (non-malignant, also referred as ‘comparison group’ or CG) and patients diagnosed with spinal Schwannian tumors, ependymomas, hemangiomas, and meningiomas (Supp. Table S1), and characterized them by transmission electron microscopy (TEM) and nanoparticle tracking analysis (NTA). EV populations that were positive for tetraspanins (‘TSPN’; CD9, CD63 and CD81 as bona fide EV markers^1,3^) and tumor-associated markers (ITGB1, CD44, CD133 and HLA class-II) were evaluated using IFCM (Fig. 1a; Supp. Table S2).

TEM analysis showed that CSF-EVs displayed the anticipated cup-shaped morphology (Fig. 1a). NTA indicated no notable variations between non-malignant CG and IST patients regarding EV dimensions (overall median size mode = 98 nm and 106.3 nm, respectively) or EV concentration per milliliter of CSF (CG median = 1.03E + 10 EVs/mL; IST median = 2.49E + 10 EVs/mL) (Fig. 1b,c). Similarly, most tumor types exhibited comparable CSF-EV concentrations when examined using IFCM (median CG and IST patients = 2.3E+06 and 8.51E + 06 per mL of CSF, respectively). As exceptions, schwannian tumors (2.02E + 07 EVs/mL CSF) and ependymoma WHO grade 1 (1.93E + 07 EVs/mL CSF) patients had 8.77- and 8.38-fold higher CSF-EV levels compared to CG (*p* < 0.05) (Fig. 1d; Supp. Table S2). Due to the antibody-washing and quality-control steps inherent to IFCM (e.g., exclusion of swarmed EVs; specific evaluation of EVs positive for CD9, CD63 and/or CD81), EV recovery and actual amount of EVs available for final analysis by IFCM is sharply reduced, when compared to bulk EV quantifications obtained by NTA. Thus, IFCM-derived concentrations should be interpreted as estimates of detectable EVs per CSF input rather than as genuine particle numbers in patient’s CSF. Despite this expected limitation differing both methodologies, the EV quantifications obtained through NTA and IFCM were positively correlated (Spearman r = 0.616; *p* < 0.0001) (Fig. 1e).

We conducted a more detailed analysis of specific single- and double-positive EV populations using IFCM. In terms of relative abundance, CD81 was the predominant TSPN present on CSF-EVs from non-malignant subjects (representing 86.34% of CG total EVs) and patients suffering from meningioma (61.7%), ependymoma WHO grade 2 (76.97%) and hemangioma (72.98%). Meanwhile, CD9 was the most abundant TSPN in patients with schwannian tumors (80.29%) and ependymoma WHO grade 1 (71.73%). In contrast, CD63 was represented only in 1.11–2.12% of CSF-EVs across all analyzed groups. Consequently, CD81^+^/CD9^+^ emerged as the primary double-positive EV subpopulation among all TSPN combinations, comprising 20.8% of CSF-EVs from non-malignant subjects and between 30.65 up to 49.06% of CSF-EVs from IST patients (Fig. 1f; Supp. Table S2).

Our analysis of TSPN^+^ EV population concentration differences between CG and IST entities revealed considerably higher levels in CSF from schwannian tumor patients. Specifically, we observed increased counts of CD9^+^ (fold change [FC] = 12.38), CD63^+^ (FC = 4.98), CD81^+^/CD9^+^ (FC = 11.97), and CD81^+^/CD63^+^ (FC = 4.33) EVs per milliliter. Furthermore, ependymoma WHO grade 1 samples exhibited elevated levels of CD9^+^ (FC = 10.56), CD63^+^ (FC = 14.03), and CD81^+^/CD9^+^ (FC = 12.77) EVs (Supp. Figure S1a–f; Supp. Table S2). The substantial log_10_ fold changes are illustrated in Supp. Figure S1g.

### CSF-EVs show divergent marker profiles between IST entities by immunophenotyping analysis

**Fig. 2:**
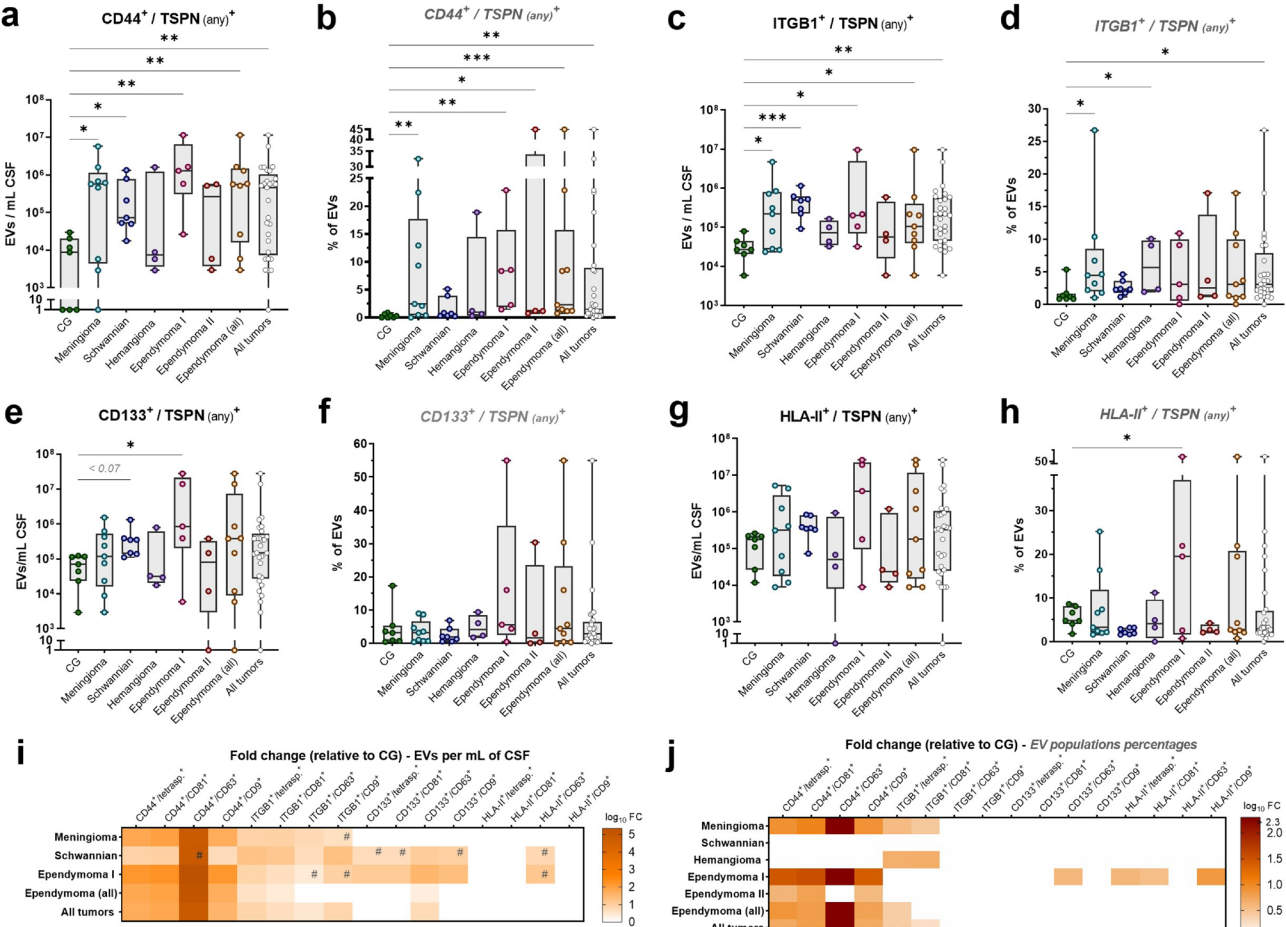
Absolute and relative levels of EV populations carrying investigated markers. CSF-EVs positive for each investigated marker and any tetraspanin (total population, in combination with CD81 and/or CD63 and/or CD9) in CG and ISTs, both at absolute (EVs per milliliter of CSF) and relative (population percentages) levels. Original (antilog) fold changes and complete statistical analysis are detailed in Supp. Table S2. (**a**) Absolute levels of total CD44^+^ EVs (regardless the tetraspanin) are significantly elevated in meningioma, schwannian tumors, ependymoma WHO grade 1, total ependymoma (WHO grades 1 and 2 together) and total IST patients (collectively), when compared with CG subjects. (**b**) Relative levels of total CD44^+^ EVs are increased in meningioma, ependymoma WHO grade 1, ependymoma WHO grade 2, total ependymoma (WHO grades 1 and 2 together) and total IST patients, than CG subjects. (**c**) Absolute levels of total ITGB1^+^ EVs are elevated in meningioma, schwannian tumors, ependymoma WHO grade 1, total ependymoma and total IST patients, than CG. (**d**) Relative levels of total ITGB1^+^ EVs are increased in meningioma, hemangioma and total IST patients, than CG. (**e**) Absolute levels of total CD133^+^ EVs are elevated in ependymoma WHO grade 1 patients, when compared with CG. A trend of significance was also observed for high levels in schwannian tumor cases. (**f**) No significant differences were observed for relative levels of total CD133^+^ EVs with any IST entity *versus* CG. (**g**) No significant differences were observed for absolute levels of total HLA-DR/DQ/DP^+^ (HLA class-II) EVs with any IST entity *versus* CG. (**h**) Relative levels of total EVs that are positive for HLA-DR/DQ/DP^+^ EVs are increased in ependymoma WHO grade 1 patients, than CG. (**i**) Log_10_ fold changes (FC) of absolute differential levels observed for CSF-EV populations (regardless the tetraspanin analyzed and also of specific double-positive subpopulations for each tetraspanin) in IST patients, as observed in panels a,c,e,g. Ependymoma WHO grade 2 and hemangioma patients are not shown in the heatmap, due to lack of significant associations. Non-significant differences were plotted with log_10_ FC = zero. (**j**) Log_10_ FC of relative differential levels observed for CSF-EV populations (regardless the tetraspanin analyzed and also of specific double-positive subpopulations for each tetraspanin) in IST patients, as observed in panels b, d, f, h. Kruskal–Wallis tests. # = *p* < 0.1; * = *p* < 0.05; ** = *p* < 0.01; *** = *p* < 0.001.

To identify potential biomarkers, we evaluated 37 surface antigens in CSF-EVs from IST patients using a multiplex EV profiling kit (Supp. Figure S2). Among these, the proteins ITGB1 (CD29), CD44, CD133 and HLA class-II (HLA-DR/DQ/DP), along with the three TSPNs (CD9, CD63, CD81), showed varied expressions across the groups (Supp. Figure S2). Based on these findings and existing literature (Supp. Table S3)^13–36^, we selected these four candidate markers for further investigation in CSF-EVs using IFCM. We then compared their absolute and relative levels between CG and IST entities (Fig. 2a–h; Supp. Table S2).

In general, EVs carrying CD44 were the most elevated population in the majority of IST patients, independently of the TSPN analyzed (in combination with CD9 and/or CD63 and/or CD81), both in absolute counts per mL of CSF and percentages of total EVs (Fig. 2a,b). After CD44, the second most relevant population consisted of ITGB1^+^ EVs (Fig. 2c,d), followed by CD133^+^ (Fig. 2e,f) and lastly by HLA class-II^+^ EVs (Fig. 2g,h). Given that TSPNs are authentic indicators of EVs and frequently associated with biogenesis and biological processes, we examined EV subsets that are positive for each marker studied, in combination with each TSPN. This ensures that the proteins under investigation are assessed on actual EV structures. Within these specific analyses, double-positive EV subpopulations were also identified at abnormal levels and showed greater potential for distinguishing IST entities. The log_10_ fold changes of all examined EV populations and specific subpopulations are depicted in Fig. 2i,j, along with their respective graph comparisons shown in Supp. Figures S3 and S4, and addressed per IST entity in the topics ahead.

#### CD44^+^/CD81^+^ EVs were the most elevated population in CSF of ependymoma patients

In comparison to non-malignant CG, CSF from ependymoma patients (WHO grades 1 and 2, collectively) showed the most pronounced differences in CD44^+^ EVs, regardless of the TSPN analyzed (Fig. 2a,b). These differences were significant for both absolute (FC = 64.7, *p* < 0.01) and relative levels (FC = 7.8, *p* < 0.001) (Fig. 2i,j; Supp. Table S2). Between both ependymoma cohorts, the contrast was most pronounced among WHO grade 1, who demonstrated 147.6-fold higher absolute levels of CD44^+^ EVs per milliliter CSF, as well as 28.9-fold higher proportions of this EV population in CSF-EVs.

**Fig. 3:**
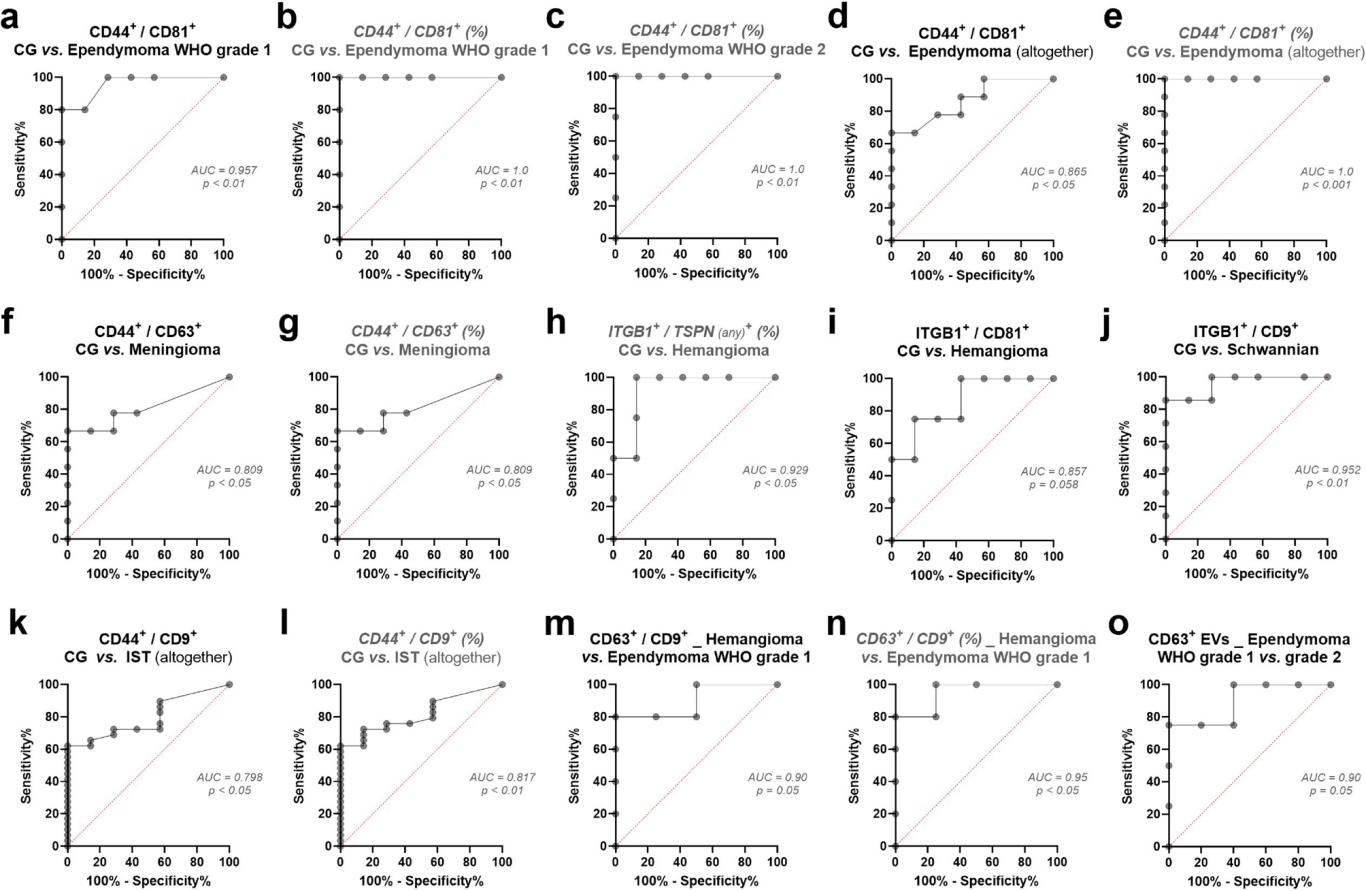
ROC analysis of discrepant EV subpopulations as biomarkers for IST detection. (**a**) Absolute levels of CD44^+^/CD81^+^ EVs discriminate CG subjects from ependymoma WHO grade 1 patients, with area under curve (AUC) greater than 95% (threshold of 247,850 EVs/mL of CSF, with 100% specificity and 80% sensitivity). (**b**, **c**) Relative levels of CD44^+^/CD81^+^ EVs discriminate CG from ependymoma WHO grade 1 and grade 2 patients, with AUC of 100%. (**d**, **e**) Absolute and relative levels of CD44^+^/CD81^+^ EVs discriminate CG from ependymoma patients (WHO grades 1 and 2 collectively). (**f**, **g**) Absolute and relative levels of CD44^+^/CD63^+^ EVs discriminate CG subjects and meningioma patients, with AUC greater than 80%. (**h**) Relative levels of total ITGB1^+^ EVs (regardless the TSPN analyzed) discriminate CG and hemangioma cases, with AUC greater than 92%. (**i**) Absolute levels of ITGB1^+^/CD81^+^ EVs showed a trend of significance for discrimination of CG and hemangioma, with AUC greater than 85%. (**j**) Absolute levels of ITGB1^+^/CD9^+^ EVs discriminate CG and schwannian tumor patients, with AUC greater than 95%. (**k**, **l**) Absolute and relative levels of ITGB1^+^/CD9^+^ EVs discriminate CG subjects from total IST patients (all analyzed collectively), with AUC greater than 79%. (**m, n**) Absolute and relative levels of CD63^+^/CD9^+^ EVs discriminate hemangioma cases from ependymoma WHO grade 1, with AUC greater than 90%. (**o**) Absolute levels of total CD63^+^ EVs discriminate ependymoma patients WHO grades 1 and 2 from each other, with AUC greater than 90%.

Notably, all three CD44^+^ double-positive EV subpopulations showed a significant increase in grade 1 ependymoma for each TSPN, both at absolute (Supp. Figure S3a–c) and relative levels (Supp. Figure S3d–f), especially for CD44^+^/CD81^+^ (FC = 196.5 and 34.5, respectively; *p* < 0.01) and CD44^+^/CD9^+^ (FC = 147.3 and 25.3, respectively; *p* < 0.01) (Supp. Table S2). ROC analysis revealed that CD44^+^/CD81^+^ EVs effectively distinguished these patients from CG (area under curve [AUC] = 95.7%, *p* < 0.01) (Fig. 3a,b).

While absolute numbers remained stable, patients with grade 2 ependymoma exhibited significant relative elevations of CD44^+^ EVs across any TSPN combination (FC = 4.12; *p* < 0.05). In particular, percentages of CD44^+^/CD81^+^ and CD44^+^/CD9^+^ subpopulations were 6.07- and 3.76-fold increased in their CSF-EVs, respectively (*p* < 0.05) (Supp. Figure S3d,e). Comparable to grade 1 findings, the relative levels of CD44^+^/CD81^+^ EVs distinguished ependymoma WHO grade 2 from CG subjects with maximal specificity and sensitivity in our limited patient cohort (AUC = 100%, *p* < 0.01) (Fig. 3c). When examining all ependymoma cases together, CD44^+^/CD81^+^ and CD44^+^/CD9^+^ emerged as the most abnormal EV subpopulations, both in absolute (FC = 86 and 48.66, respectively; *p* < 0.05) and relative (FC = 6.73 and 6.07, respectively; *p* < 0.01) terms (Supp. Figure S3a–f; Supp. Table S2). Furthermore, CD44^+^/CD81^+^ EVs proved effective in differentiating ependymoma patients from CG (Fig. 3d,e).

Besides CD44, increased levels of ITGB1^+^ and CD133^+^ EVs per milliliter of CSF were noted in patients with ependymoma WHO grade 1 (Fig. 2c,e). These elevations were particularly pronounced for the ITGB1^+^/CD81^+^ (FC = 4.72) and all three CD133^+^ EV subgroups (notably CD133^+^/CD63^+^; FC = 32.82) (Supp. Figure S3g–j; Supp. Table S2). Moreover, when analyzing all ependymoma cases together, the concentrations of ITGB1^+^/CD81^+^ and CD133^+^/CD63^+^ EVs remained higher than normal (FC = 2.61 and 2.26, *p* < 0.05).

#### CD44^+^/CD63^+^ EVs were the most elevated population in CSF of meningioma patients

In addition to ependymoma, meningioma cases also exhibited remarkably high levels of CD44^+^ EVs, both in absolute (FC = 65.8) and relative terms (FC = 8.45), independently of the TSPN examined (Fig. 2a,b).

All three CD44^+^ subpopulations were increased in meningioma CSF, with CD44^+^/CD63^+^ EVs showing the greatest elevation (FC > 1000, *p* < 0.05). Relative percentages of CD44^+^/CD63^+^ (FC > 1000) and CD44^+^/CD9^+^ (FC = 8.3) populations were also considerably higher in these patients (*p* < 0.05) (Supp. Figure S4a–e; Supp. Table S2). Consequently, both absolute and relative quantities of CD44^+^/CD63^+^ EVs effectively distinguished meningioma patients from the CG, achieving 100% specificity and 66% sensitivity (AUC = 80%, *p* < 0.05) (Fig. 3f,g).

Meningioma patients also demonstrated increased levels of ITGB1^+^ EVs per milliliter of CSF, particularly ITGB1^+^/CD63^+^ (FC = 1.13) and ITGB1^+^/CD81^+^ (FC = 8.47) EVs (Supp. Figure S4f,g). Additionally, the percentages of the ITGB1^+^/CD81^+^ subpopulation were 2.78-fold higher in their CSF-EVs (*p* < 0.01) (Supp. Figure S4h; Supp. Table S2).

#### ITGB1^+^ EVs showed significant elevation in CSF of patients with hemangioma and schwannian tumors

In the analysis of hemangioma CSF, ITGB1 emerged as the sole abnormal marker for this condition. The proportion of ITGB1^+^ EVs was 4.71 times higher in hemangioma patients’ CSF, regardless of TSPN combination (Fig. 2d), enabling differentiation from the non-malignant group (AUC = 92.9%, *p* < 0.05) (Fig. 3h). The ITGB1^+^/CD81^+^ subset was specifically linked to hemangioma presence, with 4.81-fold higher relative levels in their CSF-EVs (*p* < 0.05) (Supp. Figure S4i; Supp. Table S2). A near-significant trend (*p* < 0.06) was also noted for absolute ITGB1^+^/CD81^+^ counts in ROC analysis (Fig. 3i).

Patients with schwannian tumors displayed 18.14-fold higher concentrations of ITGB1^+^ EVs per milliliter of CSF (*p* < 0.001), irrespective of the TSPN examined (Fig. 2c). These elevations were specific to the ITGB1^+^/CD9^+^ (FC = 19.84, *p* < 0.01) and ITGB1^+^/CD81^+^ (FC = 14.16, *p* < 0.001) subpopulations (Supp. Figure S4j–l; Supp. Table S2). Furthermore, ITGB1^+^/CD9^+^ EV levels could distinguish schwannian tumor patients from CG subjects (AUC = 95.2%, *p* < 0.01) (Fig. 3j). Following ITGB1, absolute levels of CD44^+^ EVs were also elevated in schwannian tumor CSF (FC = 8.33, *p* < 0.05), as CD44^+^/CD81^+^ and CD44^+^/CD9^+^ EV subpopulations (FC = 9.5 and 5.39, respectively; *p* < 0.05) (Supp. Figure S4m,n; Supp. Table S2). Moreover, despite a non-significant trend for total CD133^+^ EVs (FC = 5.02, *p* < 0.07), abnormal absolute levels of CD133^+^/CD63^+^ EVs were observed in these patients’ CSF (FC = 11.06; *p* < 0.01) (Supp. Figure S4o).

In summary, EV populations in CSF samples show potential as clinical biomarkers for ISTs when compared to non-malignant donors.

#### Distinct EV subpopulation levels can distinguish between IST entities

Among the significant variations observed across IST entities, the counts of specific dual-positive EV subpopulations, namely ITGB1^+^/CD81^+^ (FC = 4.72), CD44^+^/CD81^+^ (FC = 68.8), and CD44^+^/CD9^+^ (FC = 34.03; relative FC = 5.04), showed the most substantial increases in the entire group of IST patients when evaluated collectively, compared to non-malignant CG (p < 0.01) (Supp. Figure S5a–d). ROC analysis revealed that both absolute and relative levels of CD44^+^/CD9^+^ EVs can differentiate IST patients from CG (AUC = 79.8% and 81.7%, respectively) (Fig. 3k,l). Consequently, these EV populations might serve as potential general biomarkers for the presence of spinal cord tumors.

Certain EV subpopulations exhibited significant differences between IST entities, making them potentially useful for classifying these tumors in relation to another. Among these comparisons, discrepant CSF-EV subpopulations were generally lower in hemangioma patients, contrasting with ependymoma WHO grade 1 cases, which displayed the highest concentrations compared to all other tumor entities (Supp. Figure S5e). Notably, CD63^+^/CD9^+^ EVs were decreased in hemangioma, particularly in comparison to ependymoma WHO grade 1. This distinction was also evident in ROC analyses for both absolute (AUC = 90%) and relative (AUC = 95%) levels (Fig. 3m,n).

Notably, absolute counts of CD63^+^ and CD133^+^/CD9^+^ EVs per milliliter of CSF were higher in ependymoma patients WHO grade 1 compared to grade 2 (FC = 24.59 and 27.79, respectively; *p* < 0.05), suggesting an utility in indicating the severity of ependymoma cases (Supp. Figure S5e; Supp. Table S2). Indeed, CD63^+^ EVs differentiated both ependymoma grades in ROC analysis (AUC = 90%) (Fig. 3o).

Compared to schwannian tumors, CSF-EVs from grade 1 ependymoma exhibited higher relative proportions of CD63^+^ (FC = 1.92; *p* < 0.01), as well as CD9^+^/CD63^+^, CD44^+^, CD44^+^/CD9^+^ and HLA class-II^+^ populations (FC = 13.25, 12, 12.61 and 9.33, respectively; *p* < 0.05). In contrast, grade 2 ependymoma showed 7.61-fold lower counts of ITGB1^+^/CD9^+^ EVs per milliliter of CSF (*p* < 0.05). Interestingly, hemangioma patients demonstrated reduced levels of various EV populations when compared to grade 1 ependymoma and other IST entities (Supp. Figure S5e; Supp. Table S2).

**Fig. 4:**
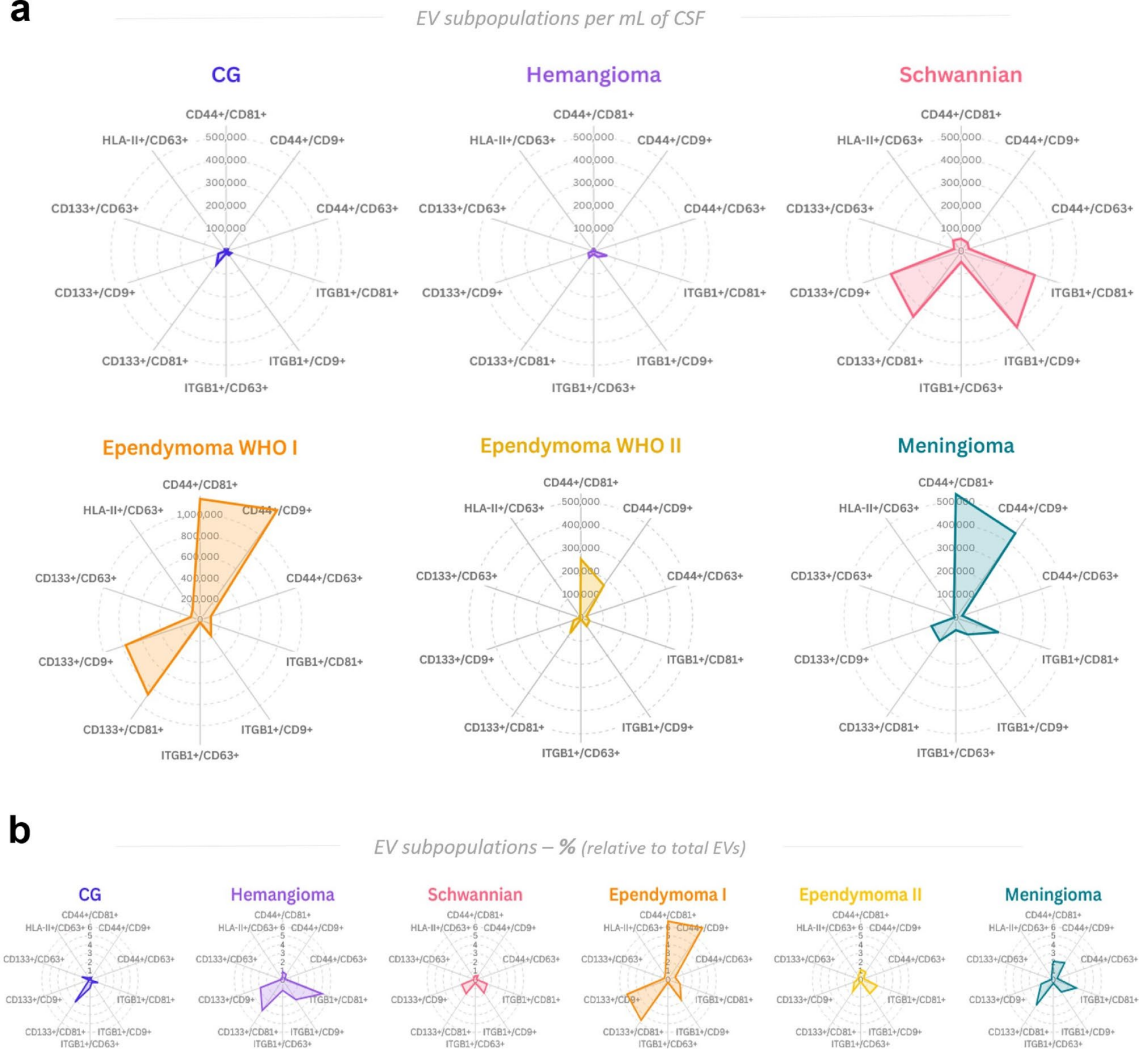
Overview of EV biomarker profiles for IST entities, according to specific double-positive EV subpopulations. (**a**) Radar chat with median values of EVs per milliliter/CSF of the aberrant EV double-positive subpopulations, as visualized in CG and ISTs. Despite the level similarities, hemangioma distinguishes from CG by increased levels of ITGB1^+^/CD81^+^ EVs. Schwannian tumor patients are characterized by elevated counts of ITGB1^+^ and CD133^+^ EVs, with moderate CD44^+^ EV counts. Meanwhile, ependymoma WHO grade 1 and 2, as well as meningioma patients, showed the most elevated levels of CD44^+^ EVs and distinguish from each other according to the CD44 combinations with tetraspanins, and also with ITGB1^+^ and CD133^+^ EV counts. (**b**) Area chart with median of percentages observed for EV subpopulations between IST entities, where relative levels of CD44^+^ EVs remain as the most relevant populations in ependymoma and meningioma cases, while ITGB1^+^ EVs remain the most frequent in hemangioma patients. Graphs created with *flourish.studio.*

To summarize, we observed distinct patterns of EV populations across different spinal tumors, which could potentially serve as biomarkers for tumor classification. Figure 4 illustrates this biomarker profiling, highlighting specific double-positive EV subpopulations that differentiate IST patients from one another (Fig. 4a,b). For instance, while hemangioma exhibits similarly low EV counts as the non-malignant group, its profile tends towards the ITGB1^+^/CD81^+^ population. In the mid-range of EV counts, schwannian tumor patients display moderate CD44^+^ and notable increases in ITGB1^+^ and CD133^+^ EV populations, whereas ependymoma WHO grade 2 shows a more specific increase in CD44^+^ EVs. At the higher end, meningioma and particularly ependymoma WHO grade 1 demonstrated the highest levels of CD44^+^ EVs, with ependymoma 1 further distinguished by significant elevations in CD133^+^ EVs (Fig. 4a). These biomarker profiles are distinctive not only in terms of absolute counts but also as relative values (Fig. 4b).

## Discussion

In recent years, there has been growing interest in investigating EVs as potential clinical biomarkers, largely due to their capacity to encase and shield molecular contents indicative of their cellular sources^1–3,37^. The challenges associated with accessing spinal tumors and the requirement for less invasive diagnostic methods^38^ make EVs particularly appealing, as they provide means to observe the tumor microenvironment through liquid biopsy and supplement conventional diagnostic approaches^39–42^. We examined EVs in CSF from patients with intradural spinal tumors, with a specific emphasis on ependymomas, schwannian tumors, meningioma, and hemangioma. For each IST type, we analyzed the prevalence of tetraspanins in conjunction with selected tumor-associated antigens (ITGB1, CD44, CD133, HLA class-II).

The varying concentrations of specific EV subpopulations in CSF across tumor types indicate that certain EV profiles can be differentiated among IST entities. Consistently with numerous studies reporting increased EV concentrations in tumors^5,9,10,37,43–46^, individuals with ependymoma WHO grade 1 and schwannian tumors showed markedly higher CSF-EV levels compared to CG. As majority of ependymoma WHO 1 cohort in this study is composed of mixopapillary ependymoma, a subtype affecting the lumbar region of the spine, it is possible that the highest EV levels are consequence of a closer contact of these tumors with the CSF. Additionally, myxopapillary ependymomas present specific histopathological features (e.g., pseudopapillary structures, abundant mucin) indicative of secretory activity^47^ and may thus also be associated with a more profusely EV release into the CSF. Meanwhile, ependymoma WHO grade 2 and especially hemangioma patients exhibited the lowest levels of EV populations herein analyzed. This could be attributed to their intramedullary location within the spine^48^, potentially encapsulating them in the cord parenchyma and limiting the diffusion of tumor-derived EVs into the CSF.

Analysis of major EV populations in CSF based on tetraspanins showed that CD81 was primarily associated with non-malignant controls, meningioma, and intramedullary tumors (ependymoma WHO grade 2 and hemangioma) in our cohort. In contrast, CD9 levels were higher in ependymoma WHO grade 1 and schwannian tumors. CD63, although the least expressed overall, also showed an increase in grade 1 ependymoma. These findings indicate tumor-specific variations in EV secretion patterns, similar to observations in other cancer types^37,45,46^. Notably, CD44^+^ EVs were the most abundant in IST patients, serving as broad indicators of IST presence, particularly for ependymoma and meningioma. CD44, a hyaluronic acid receptor and a cancer stem cell (CSC) marker, plays a role in cancer cell migration, invasion, and aggressiveness^49^. CD44⁺ tumor-associated macrophages (TAMs) have been also linked to angiogenesis in spinal ependymomas^22^. Moreover, CD44^+^ EVs were demonstrated to impact the TME by enhancing communication between tumor cells and stromal components, thereby modulating the extracellular matrix (ECM) and promoting invasion in various cancers^50–52^.

Ependymoma WHO grade 1 and 2 patients exhibited comparatively high concentrations of CD44^+^/CD81^+^ and CD44^+^/CD9^+^ EVs in CSF. CD81 is involved in vesicle trafficking and cell signaling^53^, and its association with CD44 on EV membranes has been shown to enhance cellular interactions and promote stemness and tumor proliferation^54^. The elevated levels of CD44^+^ EVs (especially CD44^+^/CD81^+^) in both absolute and relative terms might indicate the presence of tumor-initiating cells and/or TAMs involved in ependymoma pathology. The ability of CD44^+^/CD81^+^ EVs to distinguish ependymoma WHO grade 1 patients from the comparison group suggests their potential as reliable biomarkers for early diagnosis. In contrast, meningioma patients displayed an EV profile mostly composed of CD44^+^/CD63^+^ population. CD63 is highly expressed by TAMs, which are also abundant in meningioma tissues, and is typically downregulated during tumor progression^55–59^. Notably, the CD63 profile also differed between ependymoma WHO 1 and 2 grades. This divergence of EV profiles between ependymomas and meningiomas may reflect the distinct biological origins, behaviors and microenvironments of these tumor types^60–62^, thus influencing the composition and release of CD44 + EVs into the CSF.

Like CD44, CD133 has also been widely used as an indicator of CSCs (despite not exclusive to stem-like cells, with CD133⁻ populations also demonstrating tumor-initiating capacities^63^), playing a role in tumor initiation, advancement, and treatment resistance^49,64^., The increased presence of CD133^+^ EVs in ependymoma WHO grade 1 and schwannian tumors might suggest a stemness-sustaining mechanism within the microenvironment of these low-grade tumors. The notable increase of CD133^+^/CD9^+^ EVs in grade 1 ependymoma compared to grade 2 could indicate a reduction of stem-like cell populations in more advanced stages of spinal ependymomas, supporting the proposed use of CD133 for monitoring tumor progression^49,65^. This is particularly important in clinical settings, where precise patient classification based on tumor grade is crucial for assessing prognosis and determining treatment strategies^65^.

In contrast, hemangioma and schwannian tumors were distinguished more by varying levels of ITGB1^+^ EVs. ITGB1 is a crucial molecule involved in ECM adhesion, influencing cellular processes such as proliferation, survival, and migration^2,66–68^. ITGB1^+^ EVs have been associated with altered TME and stimulating angiogenesis^52,68,69^. In hemangioma and schwannian tumors, the ITGB1^+^/CD81^+^ increase may be attributed to the extensive ECM remodeling, angiogenic signaling, and highly proliferative nature of these benign tumors^70–73^. The capacity of ITGB1^+^ EVs to distinguish these patients from non-malignant CG also emphasizes their potential as diagnostic markers.

A noteworthy observation was the absence of a correlation between HLA-II^+^ EVs and IST entities in our study, prompting inquiries about the immunological microenvironment of ISTs. HLA class-II molecules typically play a role in presenting antigens to T cells and activating the adaptive immune response^74^. Unlike malignant gliomas and other CNS tumors, where HLA class-II^+^ EVs have been linked to immune evasion and T-cell response suppression^75^, the lack of significant differences in IST-associated EVs may suggest that these tumors either do not provoke a strong T-cell mediated immune response or potentially utilize alternative mechanisms for evading immune detection.

The interpretation of our findings must also be placed in the context of neuroinflammatory conditions. Studies in multiple sclerosis^76^ and other demyelinating/infectious disorders^77,78^ have reported altered EV counts and tetraspanin patterns, but these changes reflect global immune activation rather than the distinct tumor-associated antigen profiles detected here. Therefore, while some degree of diagnostic overlap with inflammatory states is possible, the panel employed in this study primarily captures tumor-relevant biology and is expected to be distinct from non-tumor immune-related signatures.

The distinct EV profiles herein identified highlight the complexity of the EV landscape in pathogenesis of ISTs and demonstrate their potential for IST classification. The detection and quantification of tumor-associated EV populations in CSF could provide a less invasive, safer, and earlier-detection method compared to current technologies. This is particularly important for meningiomas, ependymomas, and schwannian tumors, where early detection can significantly impact treatment outcomes and prognosis^2,4,11,79^, offering a way to monitor disease progression and treatment response. This approach is not intended to substitute routine imaging techniques, which remain the foundation for IST diagnosis, but rather to provide complementary biological information beyond what MRI alone can deliver.

Nevertheless, our research has limitations. For example, the low patient number, as well as the older age of our comparison group (composed of NPH cases, which may also display changes in protein composition^80^) relative to the IST cohort, reflect logistical constraints on CSF sampling in non-tumor individuals and the rarity of IST. Accordingly, we interpret group differences with caution and consider age as a potential confounder. While our findings suggest that distinct EV subpopulations in CSF could serve as indicators of IST presence and status, further studies are necessary to validate these biomarkers in larger, independent cohorts. Future research should explore the integration of these biomarkers into existing diagnostic workflows, addressing potential challenges and evaluating their practical utility in clinical settings.

To summarize, our study offers a promising path for identifying minimally invasive biomarkers to categorize and track ISTs. Through the analysis of distinct molecular patterns within CSF-EVs, we are laying the groundwork for developing more accurate and less intrusive diagnostic methods, potentially leading to improved patient outcomes in neuro-oncology. As investigations continue, we expect EV-based diagnostics and treatments to become increasingly crucial in managing spinal tumors, serving as a valuable tool for clinical decision-making and facilitating early detection and monitoring of tumor growth.

## Material and methods

### Human samples

CSF samples were obtained by lumbar puncture of adult patients suffering with intraspinal meningioma (n = 9; 77.7% grade-I), ependymoma (n = 9; 11% grade-I subependymoma, 44.4% grade-I ependymoma mixopapillar, 44.4% grade-II), hemangioma (n = 4) and schwannian tumors (n = 7; 85.7% Schwannoma grade I, 14.3% neurofibroma I plexiform), as approved by the medical ethics committee of the Chamber of Physicians in University Hospital Hamburg, and according to the Declaration of Helsinki. As a non-malignant comparison group (CG), CSF samples from individuals with normal pressure hydrocephalus (n = 7) were also enrolled in this study. Informed consent was obtained from all patients. Clinical and demographic data are shown in Supplementary Table S1.

Samples with visual appearance of blood contamination were excluded from analysis. CSF samples were pre-cleared by centrifugation to remove cell debris (300 × *g* for 7 min followed by 2000 × *g* for 10 min), and then frozen until use. For EVs enrichment, the cleared CSF samples were first centrifuged at 10,000 g for 30 min to remove large nanoparticles, and then 10 × concentrated by 4000 × *g* for 15–60 min at 4 °C, using Amicon Ultra Centrifugal Filters 10 k MWCO (Merck). The EVs retained on the filter were recovered with PBS. EVs were characterized according to the MISEV guidelines (Welsh et al., 2024), and experimental methods are reported in EV-TRACK (EV-METRIC of 57%, accession ID: EV240010)^81^.

### Transmission electron microscopy

CSF-EVs were mixed with 2% Paraformaldehyde and adsorbed to glow discharged carbon coated nickel grids (EMS 215–412-8400), washed 3 × with PBS and fixed with 1% Glutaraldehyde in PBS followed by 6 × washing in water. The grids were negatively stained with 1% uranyl acetate in water and investigated by transmission electron microscopy (TEM). TEM images were acquired with a SIS Veleta camera mounted on a FEI Tecnai G20 microscope operated at 80 kV.

### Nanoparticle tracking analysis (NTA)

For characterization of EV size and concentration, CSF-EVs were diluted in 0.22 µm filtered PBS (Dulbecco’s PBS, Gibco), and analyzed by NTA with a NanoSight LM14 instrument (Malvern, UK), equipped with a 638 nm Marlin F-033B IRF camera *(Allied Vision Technologies).* Video acquisition was performed with NTA software v3.0, using a camera level of 16. In total, 5 videos with duration of 30 s were captured per sample. Software settings for analysis were kept constant for all measurements (screen gain 6; detection threshold 4). EV sizes and concentrations per milliliter of CSF are listed in Supplementary Table S2.

### Multiplex based assay

CSF samples from IST patients were evaluated by a bead-based multiplex EV analysis by flow cytometry (MACSPlex Exosome Kit, human, Miltenyi Biotec). The samples were processed according to manufactures protocol. Briefly, CSF-EV samples had two volumes 6 µl and 60 µl and all samples were set to final volume of 120 µL using MACSPlex buffer. 15 µl of MACSPlex Exosome Capture Beads containing 39 different antibody-coated beads were added to each sample and incubated overnight on rotation at room temperature (RT) and protected from light. Beads were washed with 500 µl of MACSPlex buffer, at 3000 × g for 5 min. EV-beads complex was stained with detection antibodies (5 µl of each) anti-CD9, anti-CD63, and anti-CD81, and incubated for 1 h at RT protected from light. Next, beads were washed and incubated with 500 µl of MACSPlex buffer in rotation for 15 min, followed by centrifugation at 3000 × *g* for 5 min. The pellet of beads was resuspended and transferred to FACS tubes in final volume of 200 µl. Control tubes contained antibodies and buffer and another control tube contained beads and unstained EVs (Supp. Figure S2). Flow cytometric analysis was performed with BD LSRFortessa™ (BD Bioscences). All samples were mixed immediately before they were loaded and acquired by LSRFortessa™. Around 10,000 events was acquired for each sample.

### Imaging flow cytometry (IFCM)

IFCM was performed to analyze EV tetraspanins (CD9, CD63 and CD81) together with the selected following antigens: Integrin beta-1 (CD29), CD44, HLA-DR/DQ/DP and CD133. These antigens were selected according to Multiplex bead assay results and literature (Supp. Table S3)^13–36^. EV incubations, washings and specific procedures for data acquisition (e.g., event-rate thresholds, quality control for removal of swarmed EVs from analysis, and other technical parameters for analysis of EVs by IFCM) were described in Ricklefs et al.^5^, as herein followed with some adaptations. Briefly, CSF EVs (12 µL) were stained in 3 µL of 0.22 μm-filtered PBS containing 8% of exosome-depleted FBS (Invitrogen, cat. no. A2720801), with a cocktail of the following anti-human antibodies (3 µL each): PE-conjugated anti-CD9 (Biolegend, clone HI9a, cat. 312106), FITC-conjugated anti-CD81 (Biolegend, clone 5A6, cat. 349504), PacificBlue-conjugated anti-CD63 (Biolegend, clone H5C6, cat. 353012), and AF647-conjugated (separately): either anti-ITGB1 (Biolegend, clone TS2/16, cat. 303018), anti-CD44 (Biolegend, clone IM7, cat. 103018), anti-HLA-DR/DQ/DP (Biolegend, clone 361704, cat. 361704) or anti-CD133 (R&D systems, clone 170411, cat. FAB11331R). The 27 µL of EV-cocktail solutions were incubated for 45 min at room temperature (RT) in the dark. Stained EVs were washed with 500 μL of IFCM buffer (0.22 μm-filtered PBS containing 2% exosome-depleted FBS) using a 300 kDa filter (Nanosep, 4,000 g for 7 min, 4 °C), and resuspended in 27 μL of IFCM buffer. IFCM negative controls included cocktails without EVs and with lysed EVs (0.5% of NP40 for 30 min, RT).

Data was acquired on ImageStreamX Mark II Imaging Flow Cytometer (Amnis, Luminex Corporation), for 2 min at 60 × magnification, with low flow rate and beads removal. Fluorescent signals were detected for FITC in channel 2 (480–560 nm filter; laser voltage: 150mW), PE in channel 3 (560–595 nm; laser voltage: 100mW), PacificBlue in channel 7 (435–505 nm; laser voltage: 175 mW), and AF647 in channel 11 (642 nm; laser voltage: 100mW). All readouts were normalized to the original CSF input volume. Concentrations per milliliter of CSF of all analyzed EV subpopulations are listed the Supplementary Table S2.

### Statistical analysis

IFCM acquired data was analyzed using the IDEAS software version 6.2 (Amnis, Luminex Corporation), as previously described^5^. Objects/mL were obtained from non-swarmed values for each protein combination and corrected for input CSF volume. Absolute (EVs per mL of CSF) and relative values (percentages) of EV populations were compared among the groups by parametric (ANOVA) or non-parametric (Kruskal–Wallis) tests. EV populations whose levels were significantly different among the analyzed groups were also evaluated by ROC analysis using the Wilson-Brown method. *P* values equal or lower than 0.05 were considered significant.

## Supplementary Information

Below is the link to the electronic supplementary material.

Supplementary Material 1

Supplementary Material 2

Supplementary Material 3

Supplementary Material 4

## Data Availability

All datasets generated for this study are included in the article. Further inquiries can be directed to the corresponding author.

## References

[1] Yáñez-Mó, M. et al. Biological properties of extracellular vesicles and their physiological functions. *J. Extracell. Vesicles* **4**, 27066 (2015). 25979354 10.3402/jev.v4.27066 PMC4433489

[2] Kalluri, R. & LeBleu, V. S. The biology, function, and biomedical applications of exosomes. *Science* **367**, eaau6977 (2020). 32029601 10.1126/science.aau6977 PMC7717626

[3] Welsh, J. A. et al. Minimal information for studies of extracellular vesicles (MISEV2023): From basic to advanced approaches. *J. Extracell. Vesicles* **13**, e12404 (2024). 38326288 10.1002/jev2.12404 PMC10850029

[4] Onkar, A. et al. Smart nanoscale extracellular vesicles in the brain: Unveiling their biology, diagnostic potential, and therapeutic applications. *ACS Appl. Mater. Interfaces.* **16**, 6709–6742 (2024). 38315446 10.1021/acsami.3c16839

[5] Ricklefs, F. L. et al. Imaging flow cytometry facilitates multiparametric characterization of extracellular vesicles in malignant brain tumours. *J. Extracell. Vesicles* **8**, 1588555 (2019). 30949309 10.1080/20013078.2019.1588555 PMC6442086

[6] Osti, D. et al. Clinical significance of extracellular vesicles in plasma from glioblastoma patients. *Clin. Cancer Res.*10.1158/1078-0432.CCR-18-1941 (2019). 30287549 10.1158/1078-0432.CCR-18-1941

[7] Ricklefs, F. L. et al. Diagnostic potential of extracellular vesicles in meningioma patients. *Neuro Oncol.* **24**, 2078–2090 (2022). 35551407 10.1093/neuonc/noac127 PMC9883720

[8] Russo, M. N., Whaley, L. A., Norton, E. S., Zarco, N. & Guerrero-Cázares, H. Extracellular vesicles in the glioblastoma microenvironment: A diagnostic and therapeutic perspective. *Mol. Aspects Med.* **91**, 101167 (2023). 36577547 10.1016/j.mam.2022.101167 PMC10073317

[9] Ricklefs, F. L. et al. Circulating extracellular vesicles as biomarker for diagnosis, prognosis, and monitoring in glioblastoma patients. *Neuro Oncol.* **26**, 1280–1291 (2024). 38567448 10.1093/neuonc/noae068 PMC11226867

[10] Salviano-Silva, A. et al. Extracellular vesicles carrying tenascin-C are clinical biomarkers and improve tumor-derived DNA analysis in glioblastoma patients. *ACS Nano* **19**, 9844–9859 (2025). 40056466 10.1021/acsnano.4c13599 PMC11924321

[11] Ottenhausen, M. et al. Intradural spinal tumors in adults—Update on management and outcome. *Neurosurg. Rev.* **42**, 371–388 (2019). 29455369 10.1007/s10143-018-0957-x

[12] Zhiguo, F. et al. A swift expanding trend of extracellular vesicles in spinal cord injury research: A bibliometric analysis. *J. Nanobiotechnol.* **21**, 289 (2023). 10.1186/s12951-023-02051-6 PMC10463993 37612689

[13] Sandén, E., Eberstål, S., Visse, E., Siesjö, P. & Darabi, A. A standardized and reproducible protocol for serum-free monolayer culturing of primary paediatric brain tumours to be utilized for therapeutic assays. *Sci. Rep.* **5**, 12218 (2015). 26183281 10.1038/srep12218 PMC4505308

[14] Yu, L. et al. A clinically relevant orthotopic xenograft model of ependymoma that maintains the genomic signature of the primary tumor and preserves cancer stem cells in vivo. *Neuro Oncol.* **12**, 580–594 (2010). 20511191 10.1093/neuonc/nop056 PMC2940646

[15] Okonechnikov, K. et al. 3D genome mapping identifies subgroup-specific chromosome conformations and tumor-dependency genes in ependymoma. *Nat. Commun.* **14**, 2300 (2023). 37085539 10.1038/s41467-023-38044-0 PMC10121654

[16] Salehi, F. et al. Proteins involved in regulating bone invasion in skull base meningiomas. *Acta Neurochir.* **155**, 421–427 (2013). 23238945 10.1007/s00701-012-1577-9 PMC3569595

[17] Biswas, D. et al. Integrated meta-omics analysis unveils the pathways modulating tumorigenesis and proliferation in high-grade meningioma. *Cells* **12**, 2483 (2023). 37887327 10.3390/cells12202483 PMC10604908

[18] Tao, Y. et al. Holistic and network analysis of meningioma pathogenesis and malignancy. *BioFactors* **28**, 203–219 (2006). 17473381 10.1002/biof.5520280307

[19] Wang, X. et al. Analysis of gene expression profiling in meningioma: Deregulated signaling pathways associated with meningioma and EGFL6 overexpression in benign meningioma tissue and serum. *PLoS ONE* **7**, e52707 (2012). 23285163 10.1371/journal.pone.0052707 PMC3532066

[20] Lan, J. et al. Expression of cancer stem cell markers and their correlation with pathogenesis in vascular tumors. *Int. J. Clin. Exp. Pathol.* **8**, 12621–12633 (2015). 26722452 PMC4680397

[21] Martinez-Glez, V. et al. Meningiomas and schwannomas: Molecular subgroup classification found by expression arrays. *Int. J. Oncol.* **34**, 493–504 (2009). 19148485

[22] Zhang, Q. et al. Interrogation of the microenvironmental landscape in spinal ependymomas reveals dual functions of tumor-associated macrophages. *Nat. Commun.* **12**, 6867 (2021). 34824203 10.1038/s41467-021-27018-9 PMC8617028

[23] Aubin, R. G. et al. Pro-inflammatory cytokines mediate the epithelial-to-mesenchymal-like transition of pediatric posterior fossa ependymoma. *Nat. Commun.* **13**, 3936 (2022). 35803925 10.1038/s41467-022-31683-9 PMC9270322

[24] Yang, B., Dai, J., Pan, Y., Ma, Y. & Chu, S. Identification of biomarkers and construction of a microRNA-mRNA regulatory network for ependymoma using integrated bioinformatics analysis. *Oncol. Lett.*10.3892/ol.2019.10941 (2019). 31788082 10.3892/ol.2019.10941 PMC6865127

[25] Lewy-Trenda, I., Omulecka, A., Janczukowicz, J. & Papierz, W. CD44 expression in human meningiomas: An immunohistochemical analysis. *Pol. J. Pathol.* **55**, 33–37 (2004). 15195704

[26] Mostafa, R. R. CD44 expression in meningioma and its correlation with proliferation indices. *J. Clin. Diagn. Res.*10.7860/JCDR/2017/28438.10379 (2017). 28969134 10.7860/JCDR/2017/28438.10379 PMC5620774

[27] Abd Elhakeem, A. A. E., Essa, A. A., Soliman, R. K. & Hamdan, A. R. K. Novel evaluation of the expression patterns CD44 and MMP9 proteins in intracranial meningiomas and their relationship to the overall survival. *Egypt. J. Neurosurg.* **37**, 33 (2022).

[28] Harbi, S. et al. Infantile hemangioma originates from a dysregulated but not fully transformed multipotent stem cell. *Sci. Rep.* **6**, 35811 (2016). 27786256 10.1038/srep35811 PMC5081534

[29] Takada, S., Hojo, M., Takebe, N., Tanigaki, K. & Miyamoto, S. Stromal cells of hemangioblastomas exhibit mesenchymal stem cell-derived vascular progenitor cell properties. *Brain Tumor Pathol.* **35**, 193–201 (2018). 29936560 10.1007/s10014-018-0323-2

[30] Rehfeld, M. et al. Differential expression of stem cell markers in proliferating cells in glioma. *J. Cancer Res. Clin. Oncol.* **147**, 2969–2982 (2021). 34170383 10.1007/s00432-021-03704-5 PMC8397690

[31] Kamamoto, D., Saga, I., Ohara, K., Yoshida, K. & Sasaki, H. Association between CD133, CD44, and nestin expression and prognostic factors in high-grade meningioma. *World Neurosurg.* **124**, e188–e196 (2019). 30593958 10.1016/j.wneu.2018.12.067

[32] Yi, D. et al. Activation of PDGFR and EGFR promotes the acquisition of a stem cell-like phenotype in schwannomas. *Otol. Neurotol.* **33**, 1640–1647 (2012). 22935817 10.1097/MAO.0b013e31826a540d

[33] Griesinger, A. M. et al. Multi-omic approach identifies hypoxic tumor-associated myeloid cells that drive immunobiology of high-risk pediatric ependymoma. *iScience* **26**, 107585 (2023). 37694144 10.1016/j.isci.2023.107585 PMC10484966

[34] Donson, A. M. et al. Immune gene and cell enrichment is associated with a good prognosis in ependymoma. *J. Immunol.* **183**, 7428–7440 (2009). 19917695 10.4049/jimmunol.0902811

[35] Korshunov, A. et al. Gene expression patterns in ependymomas correlate with tumor location, grade, and patient age. *Am. J. Pathol.* **163**, 1721–1727 (2003). 14578171 10.1016/S0002-9440(10)63530-4 PMC1892422

[36] Modena, P. et al. Identification of tumor-specific molecular signatures in intracranial ependymoma and association with clinical characteristics. *J. Clin. Oncol.* **24**, 5223–5233 (2006). 17114655 10.1200/JCO.2006.06.3701

[37] van Niel, G., D’Angelo, G. & Raposo, G. Shedding light on the cell biology of extracellular vesicles. *Nat. Rev. Mol. Cell Biol.* **19**, 213–228 (2018). 29339798 10.1038/nrm.2017.125

[38] Massaad, E. et al. Predictive analytics in spine oncology research: First steps, limitations, and future directions. *Neurospine* **16**, 669–677 (2019). 31905455 10.14245/ns.1938402.201 PMC6944986

[39] Chiasserini, D. et al. Proteomic analysis of cerebrospinal fluid extracellular vesicles: A comprehensive dataset. *J. Proteomics* **106**, 191–204 (2014). 24769233 10.1016/j.jprot.2014.04.028

[40] Im, J. H. et al. Extracellular vesicles from cerebrospinal fluid of leptomeningeal metastasis patients deliver MiR-21 and induce methotrexate resistance in lung cancer cells. *Int. J. Mol. Sci.* **25**, 3124 (2024). 38542098 10.3390/ijms25063124 PMC10970033

[41] Connolly, I. D., Li, Y., Gephart, M. H. & Nagpal, S. The, “Liquid Biopsy”: the role of circulating DNA and RNA in central nervous system tumors. *Curr. Neurol. Neurosci. Rep.* **16**, 25 (2016). 26838352 10.1007/s11910-016-0629-6 PMC8423147

[42] Testa, A., Venturelli, E. & Brizzi, M. F. Extracellular vesicles as a novel liquid biopsy-based diagnosis for the central nervous system, head and neck, lung, and gastrointestinal cancers: Current and future perspectives. *Cancers* **13**, 2792 (2021). 34205183 10.3390/cancers13112792 PMC8200014

[43] Skog, J. et al. Glioblastoma microvesicles transport RNA and proteins that promote tumour growth and provide diagnostic biomarkers. *Nat. Cell Biol.* **10**, 1470–1476 (2008). 19011622 10.1038/ncb1800 PMC3423894

[44] Shao, H. et al. Protein typing of circulating microvesicles allows real-time monitoring of glioblastoma therapy. *Nat. Med.* **18**, 1835–1840 (2012). 23142818 10.1038/nm.2994 PMC3518564

[45] Wei, P. et al. Plasma extracellular vesicles detected by Single Molecule array technology as a liquid biopsy for colorectal cancer. *J. Extracell. Vesicles* **9**, 1809765 (2020). 32944195 10.1080/20013078.2020.1809765 PMC7480466

[46] Kalluri, R. & McAndrews, K. M. The role of extracellular vesicles in cancer. *Cell* **186**, 1610–1626 (2023). 37059067 10.1016/j.cell.2023.03.010 PMC10484374

[47] Jahanbakhshi, A. et al. Adjunctive treatment of myxopapillary ependymoma running head: Myxopapillary ependymoma. *Oncol. Rev.* **15**, 518 (2021). 33824699 10.4081/oncol.2021.518 PMC8018208

[48] Traul, D. E., Shaffrey, M. E. & Schiff, D. Part I: Spinal-cord neoplasms—intradural neoplasms. *Lancet Oncol.* **8**, 35–45 (2007). 17196509 10.1016/S1470-2045(06)71009-9

[49] Huang, J. L., Oshi, M., Endo, I. & Takabe, K. Clinical relevance of stem cell surface markers CD133, CD24, and CD44 in colorectal cancer. *Am. J. Cancer Res.* **11**, 5141–5154 (2021). 34765317 PMC8569346

[50] Babula, A., Gałuszka-Bulaga, A., Węglarczyk, K., Siedlar, M. & Baj-Krzyworzeka, M. CD44-hyaluronan axis plays a role in the interactions between colon cancer-derived extracellular vesicles and human monocytes. *Oncol. Lett.* **26**, 413 (2023). 37600336 10.3892/ol.2023.13999 PMC10436155

[51] Szatanek, R. & Baj-Krzyworzeka, M. CD44 and tumor-derived extracellular vesicles (TEVs). Possible gateway to cancer metastasis. *Int. J. Mol. Sci.* **22**, 1463 (2021). 33540535 10.3390/ijms22031463 PMC7867195

[52] Ekström, K. et al. Characterization of surface markers on extracellular vesicles isolated from lymphatic exudate from patients with breast cancer. *BMC Cancer* **22**, 50 (2022). 35012489 10.1186/s12885-021-08870-w PMC8744234

[53] Hamada, W. et al. Tetraspanin CD81 is expressed in human parotid cancer tissue and mediates cell proliferation. *J. Oral Maxillofac. Surg. Med. Pathol.* **36**, 300–307 (2024).

[54] Ramos, E. K. et al. Machine learning-assisted elucidation of CD81–CD44 interactions in promoting cancer stemness and extracellular vesicle integrity. *Elife* **11**, e82669 (2022). 36193887 10.7554/eLife.82669 PMC9581534

[55] Lupia, A. et al. CD63 tetraspanin is a negative driver of epithelial-to-mesenchymal transition in human melanoma cells. *J. Investig. Dermatol.* **134**, 2947–2956 (2014). 24940653 10.1038/jid.2014.258

[56] Lai, X. et al. Decreased expression of CD63 tetraspanin protein predicts elevated malignant potential in human esophageal cancer. *Oncol. Lett.* **13**, 4245–4251 (2017). 28599425 10.3892/ol.2017.6023 PMC5453118

[57] Dey, S., Basu, S. & Ranjan, A. Revisiting the role of CD63 as pro-tumorigenic or anti-tumorigenic tetraspanin in cancers and its theragnostic implications. *Adv. Biol.* **7**, 2300078 (2023). 10.1002/adbi.202300078 37142558

[58] Liu, S. et al. CD63 + tumor-associated macrophages drive the progression of hepatocellular carcinoma through the induction of epithelial-mesenchymal transition and lipid reprogramming. *BMC Cancer* **24**, 698 (2024). 38849760 10.1186/s12885-024-12472-7 PMC11157766

[59] Proctor, D. T. et al. Tumor-associated macrophage infiltration in meningioma. *Neuro-Oncol. Adv.* **1**, vdz018 (2019). 10.1093/noajnl/vdz018 PMC7212927 32642654

[60] Cerretti, G. et al. Spinal ependymoma in adults: From molecular advances to new treatment perspectives. *Front. Oncol.* **13**, 1301179 (2023). 38074692 10.3389/fonc.2023.1301179 PMC10704349

[61] Neyazi, S. et al. Transcriptomic and epigenetic dissection of spinal ependymoma (SP-EPN) identifies clinically relevant subtypes enriched for tumors with and without NF2 mutation. *Acta Neuropathol.* **147**, 22 (2024). 38265489 10.1007/s00401-023-02668-9 PMC10808175

[62] Zhou, Y. et al. Plasma extracellular vesicles proteomics in meningioma patients. *Transl. Oncol.* **47**, 102046 (2024). 38943923 10.1016/j.tranon.2024.102046 PMC11261147

[63] Moreno-Londoño, A. P. & Robles-Flores, M. Functional roles of CD133: More than stemness associated factor regulated by the microenvironment. *Stem Cell Rev. Rep.* **20**, 25–51 (2024). 37922108 10.1007/s12015-023-10647-6 PMC10799829

[64] Glumac, P. M. & LeBeau, A. M. The role of CD133 in cancer: A concise review. *Clin. Transl. Med.* **7**, 18 (2018). 29984391 10.1186/s40169-018-0198-1 PMC6035906

[65] Brocco, D. et al. Blood circulating CD133+ extracellular vesicles predict clinical outcomes in patients with metastatic colorectal cancer. *Cancers* **14**, 1357 (2022). 35267665 10.3390/cancers14051357 PMC8909146

[66] Desgrosellier, J. S. & Cheresh, D. A. Integrins in cancer: Biological implications and therapeutic opportunities. *Nat. Rev. Cancer* **10**, 9–22 (2010). 20029421 10.1038/nrc2748 PMC4383089

[67] Liu, F., Wu, Q., Dong, Z. & Liu, K. Integrins in cancer: Emerging mechanisms and therapeutic opportunities. *Pharmacol. Ther.* **247**, 108458 (2023). 37245545 10.1016/j.pharmthera.2023.108458

[68] DeRita, R. M. et al. Tumor-derived extracellular vesicles require β1 integrins to promote anchorage-independent growth. *iScience* **14**, 199–209 (2019). 30981115 10.1016/j.isci.2019.03.022 PMC6461598

[69] Lv, T. et al. Cancer-associated fibroblast-derived extracellular vesicles promote lymph node metastases in oral cavity squamous cell carcinoma by encapsulating ITGB1 and BMI1. *BMC Cancer* **24**, 113 (2024). 38254031 10.1186/s12885-024-11855-0 PMC10804601

[70] Kato, K., Teferi, N., Challa, M., Eschbacher, K. & Yamaguchi, S. Vertebral hemangiomas: A review on diagnosis and management. *J. Orthop. Surg. Res.* **19**, 310 (2024). 38789994 10.1186/s13018-024-04799-5 PMC11127296

[71] Wang, L.-J., Zou, H.-M., Hou, F., Wang, G.-X. & Gao, C.-P. Aggressive vertebral hemangiomas contain no adipose tissue resulting in thoracic spine kyphosis: A case report. *Medicine* **103**, e37885 (2024). 38640290 10.1097/MD.0000000000037885 PMC11029966

[72] Lenzi, J. et al. Spinal nerves schwannomas: Experience on 367 cases—Historic overview on how clinical, radiological, and surgical practices have changed over a course of 60 years. *Neurol. Res. Int.* **2017**, 1–12 (2017). 10.1155/2017/3568359 PMC5624174 29075532

[73] Nguyen, H. T. N. et al. Matrix metalloproteinase 9: An emerging biomarker for classification of adherent vestibular schwannoma. *Neuro-Oncol. Adv.* **6**, vdae058 (2024). 10.1093/noajnl/vdae058 PMC11181934 38887507

[74] Roche, P. A. & Furuta, K. The ins and outs of MHC class II-mediated antigen processing and presentation. *Nat. Rev. Immunol.* **15**, 203–216 (2015). 25720354 10.1038/nri3818 PMC6314495

[75] Qian, J. et al. TLR2 promotes glioma immune evasion by downregulating MHC class II molecules in microglia. *Cancer Immunol. Res.* **6**, 1220–1233 (2018). 30131377 10.1158/2326-6066.CIR-18-0020

[76] Anandan, S. et al. Brain-derived blood biomarkers in multiple sclerosis—current trends and beyond. *Front. Immunol.* **16**, 1569503 (2025). 40589765 10.3389/fimmu.2025.1569503 PMC12206755

[77] Pleet, M. L. et al. Viral Immune signatures from cerebrospinal fluid extracellular vesicles and particles in HAM and other chronic neurological diseases. *Front. Immunol.* **14**, 1235791 (2023). 37622115 10.3389/fimmu.2023.1235791 PMC10446883

[78] Cruz, C. G., Sodawalla, H. M., Mohanakumar, T. & Bansal, S. Extracellular vesicles as biomarkers in infectious diseases. *Biology* **14**, 182 (2025). 40001950 10.3390/biology14020182 PMC11851951

[79] Yang, L. et al. DNA of neutrophil extracellular traps promotes cancer metastasis via CCDC25. *Nature* **583**, 133–138 (2020). 32528174 10.1038/s41586-020-2394-6

[80] Kamalian, A. et al. Molecular signatures of normal pressure hydrocephalus: A large-scale proteomic analysis of cerebrospinal fluid. *Fluids Barr. CNS* **21**, 64 (2024). 10.1186/s12987-024-00561-5 PMC11312837 39118132

[81] Van Deun, J. et al. EV-TRACK: Transparent reporting and centralizing knowledge in extracellular vesicle research. *Nat. Methods*10.1038/nmeth.4185 (2017). 28245209 10.1038/nmeth.4185

