# Supplementary material for: Profiling extracellular vesicles from cerebrospinal fluid for classification of intradural spinal tumors
Source: Sci Rep. 2025 Oct 20;15:36479. doi: 10.1038/s41598-025-23190-w (PMC12537865; doi:10.1038/s41598-025-23190-w)
Supplement: Supplementary file 4 — Supplementary Material 4 [file 41598_2025_23190_MOESM4_ESM.docx]

## SUPPLEMENTARY FIGURES

**
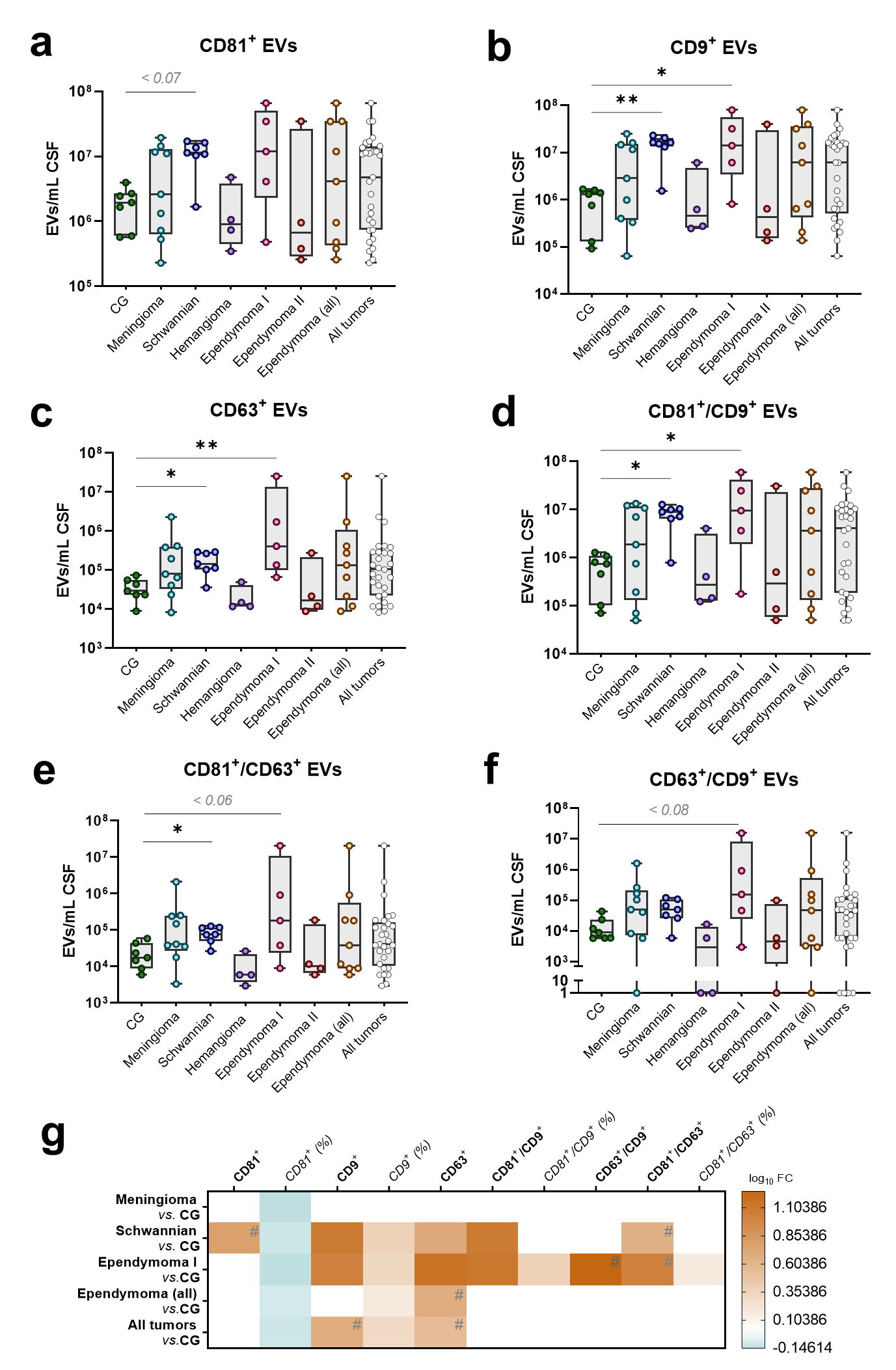
**

**Supp. Figure S1. CSF-EV concentration levels per tetraspanin populations.**

**(a)** Positive EVs for CD81 tetraspanin in CSF of IST patients. No significant differences were observed for any tumor entity, despite a trend (p < 0.07) of 5.8-fold higher levels of CD81^+^ EVs per milliliter of CSF in patients with Schwannian tumors (Kruskal-Wallis test).

**(b)** Positive EVs for CD9 tetraspanin are 12.38- and 10.56-fold elevated in CSF samples from schwannian tumor and ependymoma WHO grade 1 patients, respectively, when compared to CG subjects (Kruskal-Wallis test; * = p < .05; ** = p < 0.01).

**(c)** Positive EVs for CD63 tetraspanin are 4.98- and 14.03-fold more concentrated in CSF from schwannian and ependymoma WHO grade 1 patients, respectively, when compared to CG subjects (Kruskal-Wallis test; * = p < .05; ** = p < 0.01).

**(d)** Dual-positive EVs for CD81 and CD9 tetraspanins are 11.97- and 12-77-fold elevated in CSF samples from schwannian and ependymoma WHO grade 1 patients, respectively, when compared to CG (Kruskal-Wallis test; * = p < .05).

**(e)** Dual-positive EVs for CD81 and CD63 tetraspanins are 4.33-fold increased in CSF samples from schwannian patients than in CG subjects (Kruskal-Wallis test; * = p < .05). A trend of 10.37-fold higher levels (p < 0.06) was also observed for ependymoma WHO grade 1 cases.

**(f)** Dual-positive EVs for CD63 and CD9 tetraspanins were not significantly different in any tumor entity, despite a trend (p < 0.07) of significant 17.82-fold higher levels in ependymoma WHO grade 1 patients (p < 0.08) (Kruskal-Wallis test).

**(g)** Log_10_ fold changes (FC) of CSF-EV subpopulations whose levels were significantly different in spinal-tumor patients, as observed in panels A-F. Non-significant differences were plotted with log_10_ FC = zero. # = significance trend (0.05 < p < 0.01).


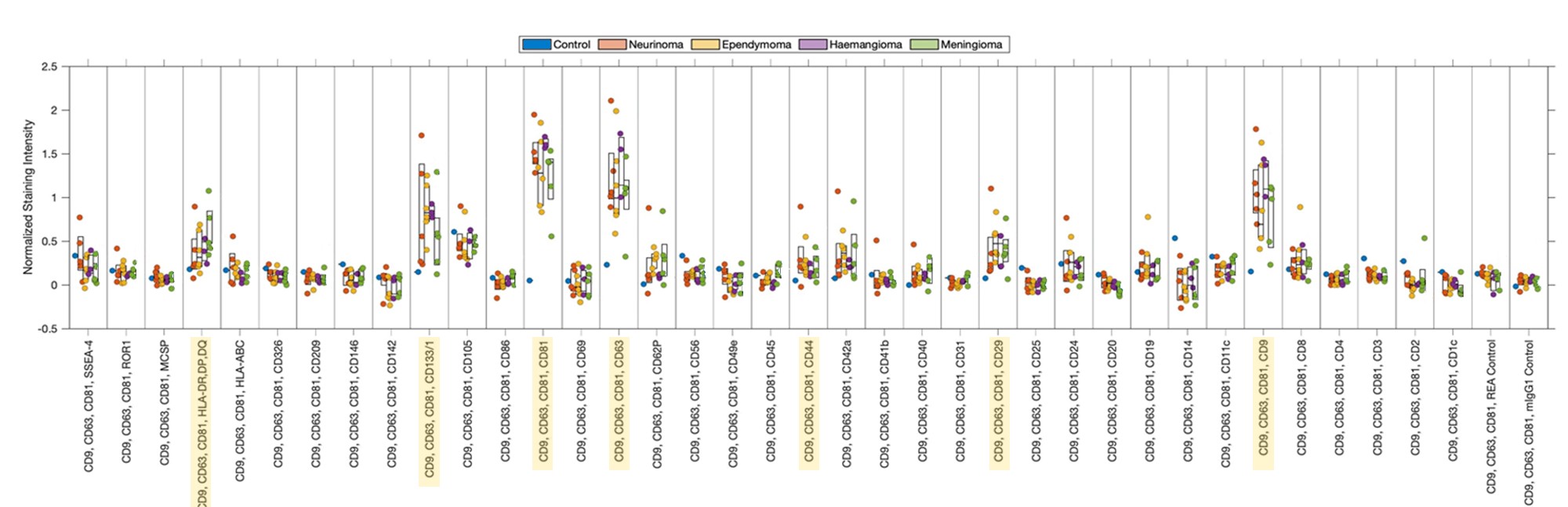


**Supp. Figure S2. Multiplex analysis of CSF-EVs.**

MACSPlex multiplex analysis of 37 antigens in CSF-EVs of different tumor entities. The membrane proteins CD29 (ITGB1), CD44, CD133 and HLA-DR/DQ/DP (HLA-II), in addition to the tetraspanins (CD81, CD9 and CD63) were observed with more divergent expressions among the groups (highlighted in yellow). Due to this fact, and also in accordance to the literature, these antigens were selected to be investigated by IFCM.


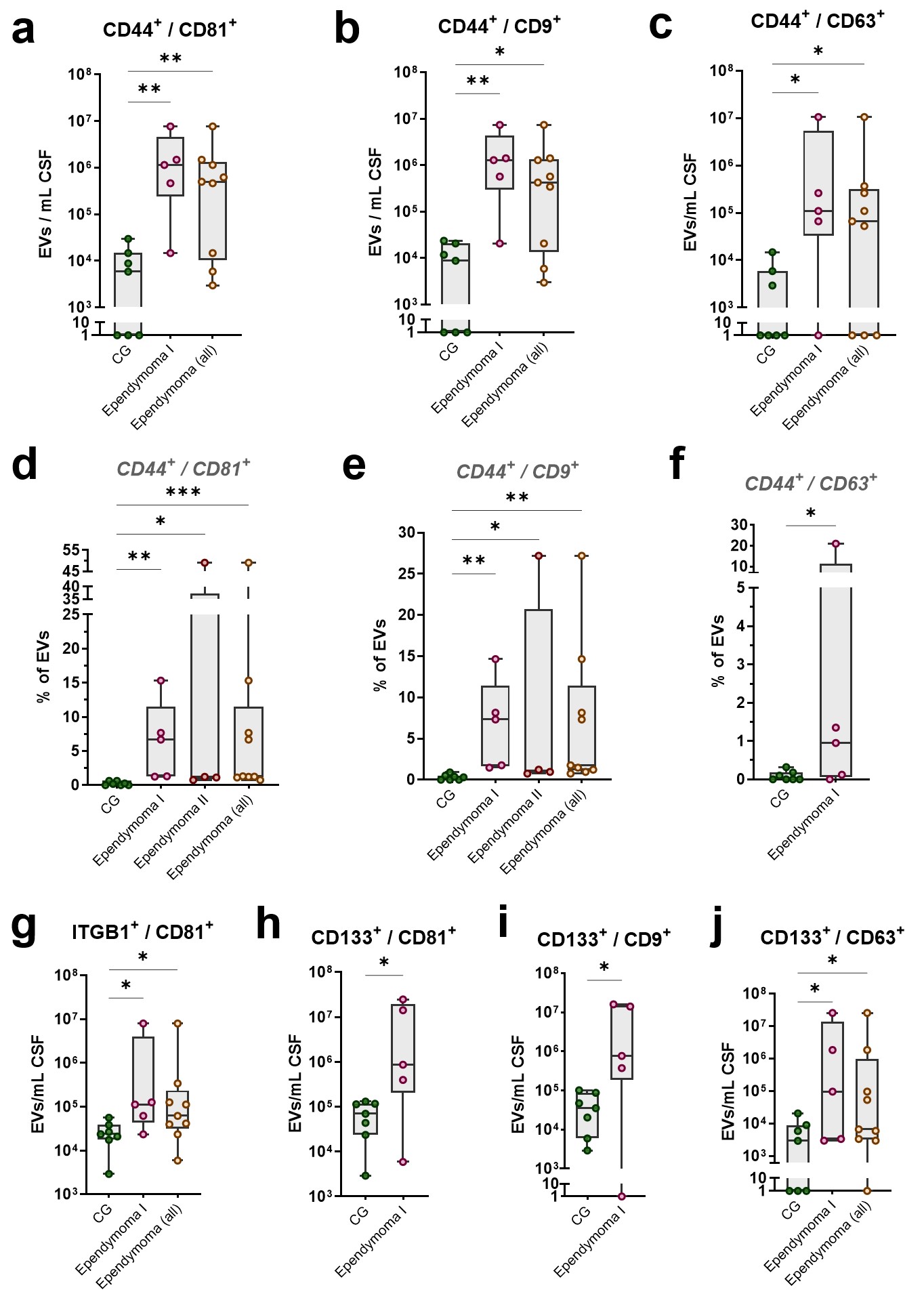


**Supp. Figure S3. Analysis of EV populations in CSF of ependymoma patients.**

Significant differences of double-positive CSF-EV subpopulations (investigated markers in combination with specific tetraspanins) in ependymoma patients, both at absolute and relative levels, when compared to CG subjects. Statistical results were obtained by Kruskal-Wallis analysis and are detailed in Supp. Table S2.

**(a)** Absolute concentrations of CD44^+^/CD81^+^ EVs are 196.53- and 86.01-fold elevated in CSF from ependymoma WHO grade 1 and total ependymoma (WHO grades 1 and 2 together) patients, respectively, in comparison to CG.

**(b)** Absolute concentrations of CD44^+^/CD9^+^ EVs are 147.27- and 48.66-fold elevated in CSF from ependymoma WHO grade 1 and total ependymoma patients, respectively.

**(c)** Absolute concentrations of CD44^+^/CD63^+^ EVs are 219510- and 132200-fold elevated in CSF from ependymoma WHO grade 1 and total ependymoma patients, respectively.

**(d)** Relative levels of CD44^+^/CD81^+^ population are 34.5-, 6.07- and 6.73-fold increased in CSF-EVs from ependymoma WHO grade 1, ependymoma WHO grade 2 and total ependymoma patients, respectively.

**(e)** Relative levels of CD44^+^/CD9^+^ population are 25.29-, 3.76- and 6.07-fold increased in CSF-EVs from ependymoma WHO grade 1, ependymoma WHO grade 2 and total ependymoma patients, respectively.

**(f)** Relative levels of CD44^+^/CD63^+^ population are 12396-fold increased in CSF-EVs from ependymoma WHO grade 1 patients.

**(g)** Absolute concentrations of ITGB1^+^/CD81^+^ EVs are 4.72- and 2.61-fold elevated in CSF from ependymoma WHO grade 1 and total ependymoma patients.

**(h)** Absolute concentrations of CD133^+^/CD81^+^ EVs are 12.39-fold increased in CSF-EVs from ependymoma WHO grade 1 patients.

**(i)** Absolute concentrations of CD133^+^/CD9^+^ EVs are 21.85-fold increased in CSF-EVs from ependymoma WHO grade 1 patients.

**(j)** Absolute concentrations of CD133^+^/CD63^+^ EVs are 32.82- and 2.26-fold elevated in CSF from ependymoma WHO grade 1 and total ependymoma patients, respectively.

* = p < 0.05; ** = p < 0.01; *** = p < 0.001.


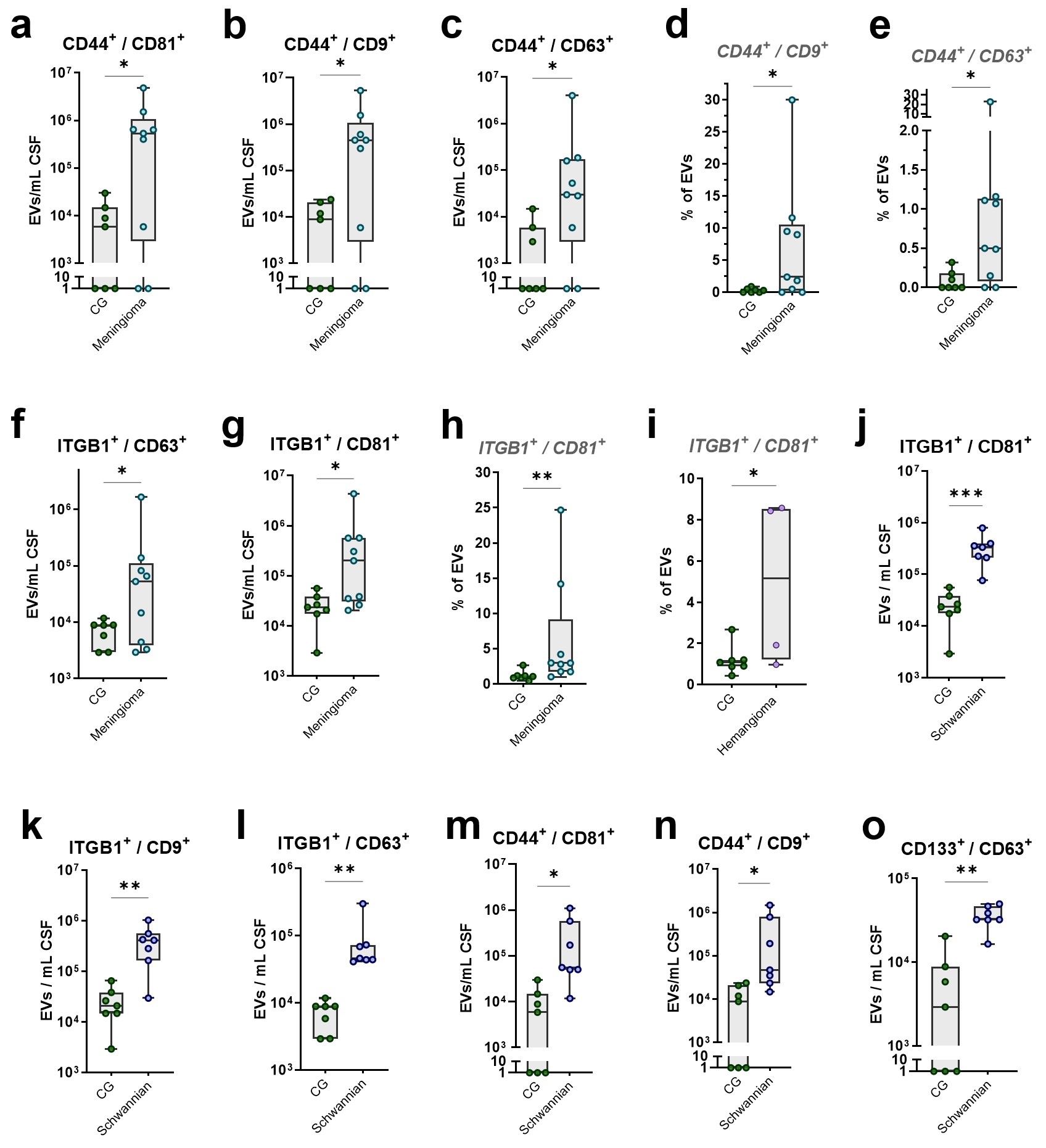


**Supp. Figure S4. Analysis of EV populations in CSF of meningioma, hemangioma and schwannian tumor patients.**

Significant differences of double-positive CSF-EV subpopulations (investigated markers in combination with specific tetraspanins) in patients with spinal meningioma, hemangioma and schwannian tumors, both at absolute and relative levels, when compared to CG subjects. Statistical results were obtained by Kruskal-Wallis analysis and are detailed in Supp. Table S2.

**(a)** Absolute concentrations of CD44^+^/CD81^+^ EVs are 91.45-fold elevated in CSF from meningioma patients.

**(b)** Absolute concentrations of CD44^+^/CD9^+^ EVs are 51.54-fold elevated in CSF from meningioma patients.

**(c)** Absolute concentrations of CD44^+^/CD63^+^ EVs are 59641-fold elevated in CSF from meningioma patients.

**(d)** Relative levels of CD44^+^/CD9^+^ population are 8.27-fold increased in CSF-EVs from meningioma patients.

**(e)** Relative levels of CD44^+^/CD63^+^ population are 6502-fold increased in CSF-EVs from meningioma patients.

**(f)** Absolute concentrations of ITGB1^+^/CD63^+^ population are 5.98-fold elevated in CSF-EVs from meningioma patients.

**(g)** Absolute concentrations of ITGB1^+^/CD81^+^ EVs are 8.47-fold elevated in CSF from meningioma patients.

**(h)** Relative levels of ITGB1^+^/CD81^+^ population are 2.78-fold increased in CSF-EVs from meningioma patients.

**(i)** Relative levels of ITGB1^+^/CD81^+^ population are 4.81-fold increased in CSF-EVs from hemangioma patients.

**(j)** Absolute concentrations of ITGB1^+^/CD81^+^ EVs are 14.16-fold elevated in CSF from patients with schwannian tumors.

**(k)** Absolute concentrations of ITGB1^+^/CD9^+^ EVs are 19.84-fold elevated in CSF from patients with schwannian tumors.

**(l)** Absolute concentrations of ITGB1^+^/CD63^+^ EVs are 5.23-fold elevated in CSF from patients with schwannian tumors.

**(m)** Absolute concentrations of CD44^+^/CD81^+^ EVs are 9.5-fold elevated in CSF from patients with schwannian tumors.

**(n)** Absolute concentrations of CD44^+^/CD9^+^ EVs are 5.39-fold elevated in CSF from patients with schwannian tumors.

**(o)** Absolute concentrations of CD133^+^/CD63^+^ EVs are 11.06-fold elevated in CSF from patients with schwannian tumors.

* = p < 0.05; ** = p < 0.01; *** = p < 0.001


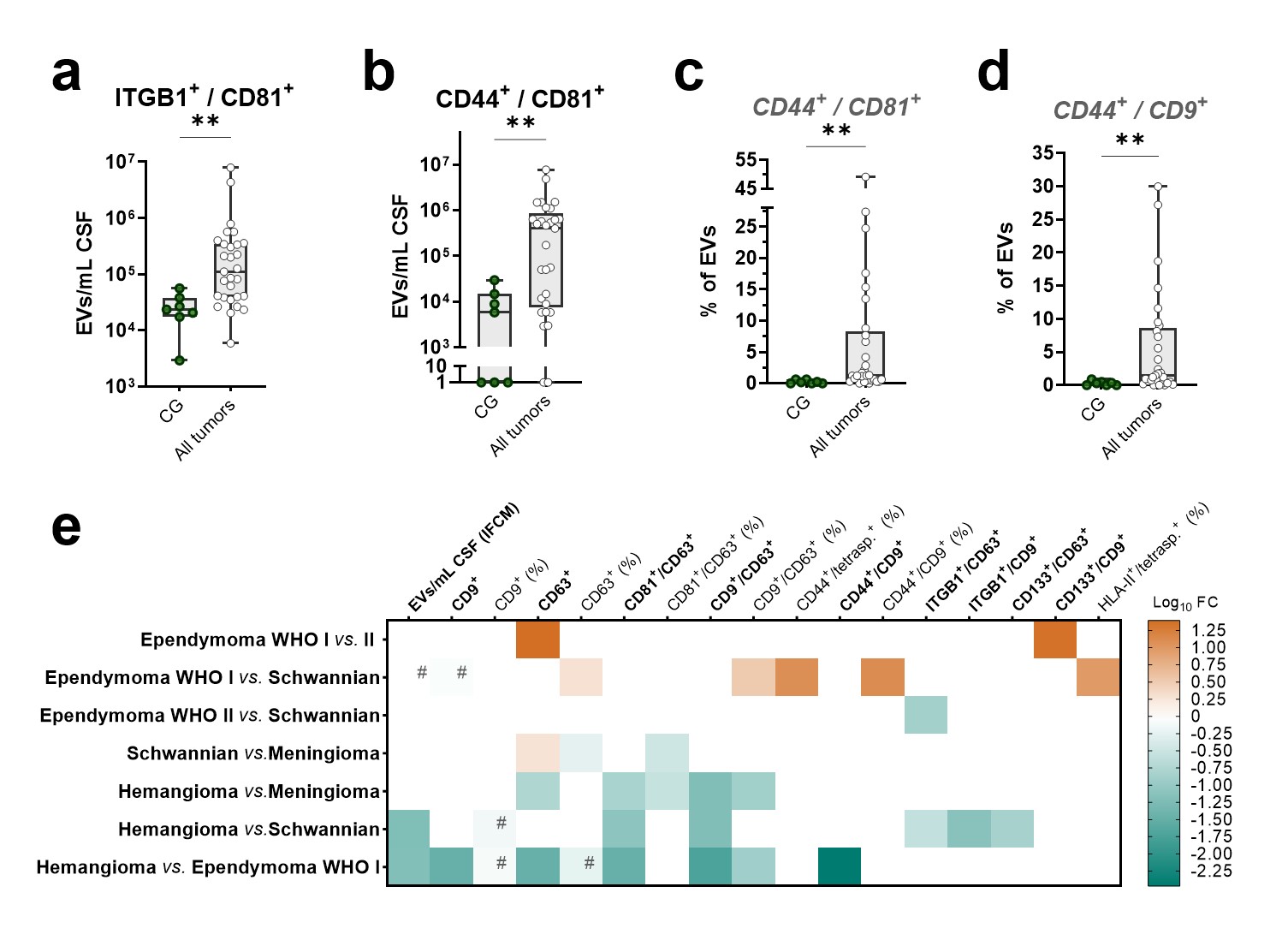


**Supp. Figure S5. EV subpopulations as IST indicators and for discrimination between tumor entities.**

Significant differences of double-positive CSF-EV subpopulations (investigated markers in combination with specific tetraspanins) in ependymoma patients, both at absolute and relative levels, when compared to CG subjects. Statistical results were obtained by Kruskal-Wallis analysis and are detailed in Supp. Table S2.

**(a)** Absolute levels of the specific dual-positive EV subpopulation ITGB1^+^/CD81^+^ are 4.72-fold elevated in total IST patients, when compared with CG.

**(b)** Absolute levels of the dual-positive EV subpopulation CD44^+^/CD81^+^ are 68.77-fold elevated in total IST patients, when compared with CG.

**(c)** Relative levels of the dual-positive EV subpopulation CD44^+^/CD81^+^ are 6.5-fold increased in total IST patients, when compared with CG.

**(d)** Relative levels of the dual-positive EV subpopulation CD44^+^/CD9^+^ are 5.04-fold increased in total IST patients, when compared with CG.

**(e)** Log_10_ FC of significant differences observed for CSF-EV populations, both at absolute and relative levels, when IST entities are compared with each other. Non-significant differences are represented as FC = zero.

# = 0.07 < p < 0.1; * = p < 0.05; ** = p < 0.01; *** = p < 0.001.
